# Profiling steroid hormone landscape of bladder cancer reveals depletion of intratumoural androgens to castration levels: a cross-sectional study

**DOI:** 10.1016/j.ebiom.2024.105359

**Published:** 2024-09-28

**Authors:** Kimmo Kettunen, Julia Mathlin, Tarja Lamminen, Asta Laiho, Merja R. Häkkinen, Seppo Auriola, Laura L. Elo, Peter J. Boström, Matti Poutanen, Pekka Taimen

**Affiliations:** a Institute of Biomedicine and FICAN West Cancer Centre, University of Turku, Turku, Finland; b Turku Bioscience Centre, University of Turku and Åbo Akademi University, Turku, Finland; c Department of Urology and FICAN West Cancer Centre, Turku University Hospital, and University of Turku, Turku, Finland; d Department of Health Security, Finnish Institute for Health and Welfare, Kuopio, Finland; e School of Pharmacy, University of Eastern Finland, Kuopio, Finland; f Department of Internal Medicine and Clinical Nutrition, Institute of Medicine, Sahlgrenska Academy at University of Gothenburg, Gothenburg, Sweden; g Turku Center for Disease Modeling, University of Turku, Turku, Finland; h Department of Pathology, Turku University Hospital, Turku, Finland

**Keywords:** Bladder cancer, Sex steroids, Androgens, Steroid profile, Steroidomics

## Abstract

**Background:**

Bladder cancer is a highly over-represented disease in males. The involvement of sex steroids in bladder carcinogenesis and the utilisation of steroid hormone action as a therapeutic target have been frequently proposed. However, the intratumoural steroid milieu remains unclear.

**Methods:**

We used mass spectrometry and transcriptomic profiling to determine the levels of 23 steroid hormones and the expression of steroidogenic enzymes in primary tumours from patients who underwent transurethral resection (n = 24), and tumours and adjacent morphologically benign bladder tissues from treatment-naïve patients, who underwent radical cystectomy (n = 20). The corresponding steroids were determined from the patients’ sera.

**Findings:**

Our results show that both bladder tumours and non-tumour tissues are androgen-poor, with DHT being virtually unquantifiable and testosterone at castration levels. Intratumoural enzymes that inactivate potent androgens (e.g., *HSD17B2*) exhibited similar tumour aggressiveness-linked downregulation, as reported in advanced forms of classical steroid-dependent cancers, whereas there was little change in the corresponding activating enzymes. Finally, our results suggest cancer aggressiveness-linked dissimilarities in steroid profiles; the patients with overall low circulating steroid levels and those with an association between androgen receptor expression and intratumoural testosterone levels in place had fewer recurrences than the rest.

**Interpretation:**

By revealing the steroid landscape of bladder cancer, our study not only underscores the androgen-poor nature of the malignancy but also identifies potential alterations in steroid profiles that are linked to disease aggressiveness.

**Funding:**

The 10.13039/501100010711Cancer Foundation Finland, the Finnish State Research Funding (VTR).

**Research in context**

**Evidence before this study**

There are significant sex disparities in the incidence and characteristics of bladder cancer, even after adjusting for smoking and occupational factors. Various biological factors are thought to contribute to these disparities, such as differences in the immune system, metabolism, or hormonal environment. Androgen action has been associated with bladder cancer development in animal models. Moreover, men tend to have luminal papillary tumours with active androgen signalling, whereas women often present with higher-stage disease and rare histological subtypes. Finally, 5a-reductase inhibitors and androgen deprivation therapy have been associated with reduced recurrence rates, particularly in low-grade disease.

**Added value of this study**

We investigated the intratumoural and systemic steroid profiles of patients with various grades and stages of the bladder cancer using a panel of 23 steroid precursors and hormones. We observed the androgen environment of tumours and benign bladder tissue to be on par with castration-treated prostatic tissue. Furthermore, the stage-linked expression profiles of steroid metabolising enzymes were similar to those described in advanced forms of prostate cancer. Finally, patients with non-invasive disease and low systemic steroid levels had fewer recurrences during the follow-up period.

**Implications of all the available evidence**

Our results bolster the hypothesis that androgens promote bladder cancer development. Since bladder cancer arises in an androgen-poor environment, the direct effects of steroid biosynthesis inhibitors against already developed cancer cells are likely limited. However, they may be beneficial in the secondary prevention of the disease, and further studies with larger patient cohorts are warranted.

## Introduction

Male sex is a significant risk factor for bladder cancer, with a three-to-four-fold higher incidence in men compared to women.^1^^,^^2^ In contrast, women are more often diagnosed with higher-stage diseases and rare histological subtypes.^3^^,^^4^ Discrepancies in the sex steroid milieu, immune system, and genetic factors are suggested to influence these sex-linked disparities.^3^^,^^5^

Preclinical and clinical studies have demonstrated the involvement of androgen action in bladder cancer carcinogenesis.^6^ For example, in a chemically induced model, AR-knockout mice did not develop bladder tumours without DHT supplementation, and castrated mice developed fewer tumours than the controls.^7^ Moreover, supplemented androgens have been shown to promote the proliferation and migration of bladder cancer cells.^8^, ^9^, ^10^ In clinical specimens, 13–55% of tumours are AR-positive, and its expression is associated with low-grade tumours and improved recurrence-free survival.^3^^,^^11^ The use of 5α-reductase inhibitors (5-ARIs) and ADT is also associated with a reduced recurrence and progression rate, especially in low-grade non-muscle-invasive bladder cancer (NMIBC); however, their effect on the incidence is unclear.^12^^,^^13^ Recently, finasteride has been suggested to improve the overall survival of patients with bladder cancer, specifically in patients with NMIBC.^14^ Finally, the molecular subtypes of bladder cancer appear to be associated with sex, as men more frequently have luminal papillary tumours with active androgen signalling, whereas women have more basal tumours and higher immune activity.^15^

Oestrogen receptor (ER) signalling is also posited to play a role in bladder cancer. ERα-mediated signalling is associated with the inhibition of oncogenesis, whereas ERβ signalling promotes it.^16^ However, their actual prognostic significance remains inconsistent.^17^ The findings of epidemiological studies are likewise inconclusive, and the impact of female hormonal and reproductive factors varies among studies.^18^, ^19^, ^20^

Previous studies found no relationship between serum testosterone levels and tumour characteristics,^21^ but low levels of some androgen precursors (DHEA and DHEAS) were associated with the risk of bladder cancer.^22^ However, local activation or inactivation of steroid hormones may alter intratumoural steroid levels, making them inconsistent with the serum levels. Therefore, examining the intratumoural steroidogenic environment and accurately determining actual ligand quantities is necessary. To date, the intratumoural steroid milieu of bladder cancer is largely unknown, and a better understanding of this milieu and its relationship with circulating steroid levels is warranted, as such data could favour the use of steroid biosynthesis inhibitors in therapy or prevention. As well-known drugs, 5-ARIs are an especially intriguing option, although their cancer-suppressive benefit may be limited mainly to early-stage disease.^12^^,^^23^, ^24^, ^25^

In the current study, we investigated intratumoural steroid profiles of bladder cancer and compared them with those of adjacent non-cancerous bladder tissue and patient sera. To identify undisclosed alterations in the steroidogenic environment at various stages of disease progression, we comprehensively analysed the steroid levels in high- and low-grade tumours and further examined how the expression of steroid-metabolising enzymes is associated with them. Our results shed new light on the intricate interactions between steroids and tumour progression, which may have important implications for future therapeutic approaches for bladder cancer.

## Methods

### Patient cohorts

**Fig. 1:**
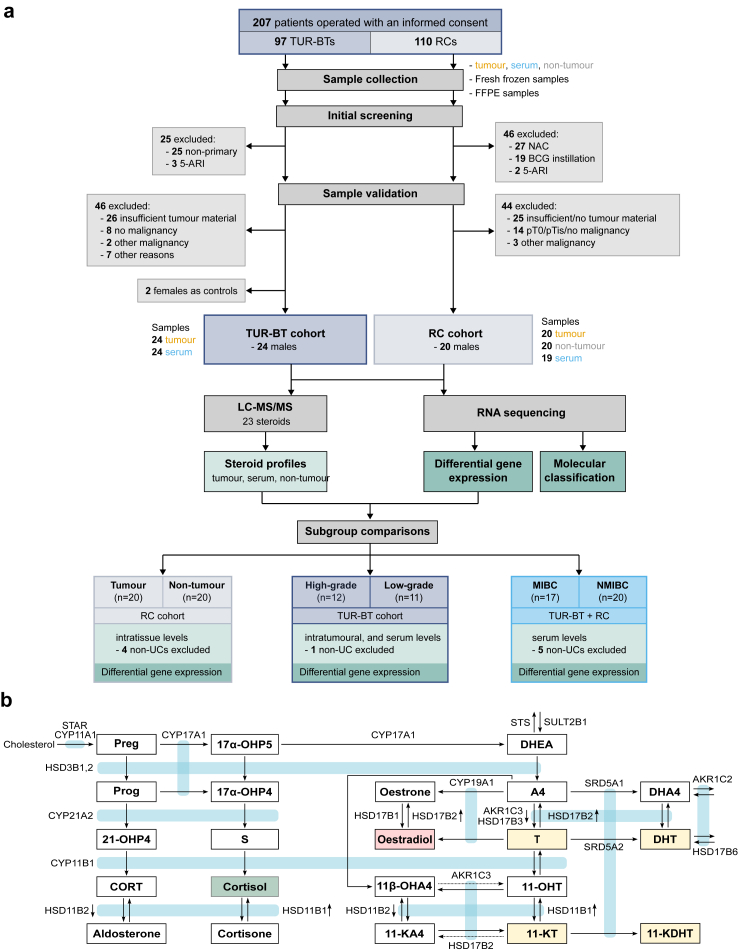
**Study design**. **a** Flow chart of the study's sample selection, performed analysis, and comparisons. **b** Flow chart of the measured steroid hormones and the most significant known steroidogenic enzymes metabolising them.

Fresh tissue and serum samples were prospectively collected from patients who underwent transurethral resection (TUR-BT) or radical cystectomy (RC) due to bladder cancer. Following initial screening and sample validation, we included 24 TUR-BT patients with primary tumours (TUR-BT cohort) and 20 RC patients without neo-adjuvant chemotherapy (NAC) or prior BCG treatment (RC cohort) for further analysis. Overlapping patients between the cohorts were avoided, and the users of 5-ARI medication were excluded from the study. In addition, we collected control samples from two post-menopausal females who underwent TUR-BT. All patients underwent surgery at Turku University Hospital between November 2013 and January 2020, and survival data were collected in July 2024. An overview of the study is shown in Fig. 1a, and a summary of the patient and tumour characteristics is provided in Table 1.

**Table 1:** Patient and tumour characteristics of the RC and TUR-BT cohorts.

Variable	RC, n = 20	TUR-BT, n = 24
Median age, yr (IQR)	72 (59, 78)	66 (62, 73)
Smoking, *n* (%)		
Active	9 (50%)	12 (50%)
Former	7 (39%)	10 (42%)
Never	2 (11%)	2 (8%)
NA	2	0
Survival, *n* (%)		
Alive, NED^a^	3 (15%)	10 (42%)
Alive, recurrence^b^	2 (10%)	8 (33%)
Died due to bladder cancer	11 (55%)	3 (13%)
Died, other cause	3 (15%)	3 (13%)
Lost to follow-up^c^	1 (5%)	0 (0%)
pT stage, *n* (%)		
pTa	2 (10%)	14 (58%)
pT1	0 (0%)	6 (25%)
pT2	3 (15%)	4 (17%)
pT3	13 (65%)	–
pT4	2 (10%)	–
pN stage, *n* (%)		
pN0/Nx	10 (50%)	24 (100%)
pN+	10 (50%)	–
ISUP grade, *n* (%)		
High-grade	18 (100%)	12 (52%)
Low-grade	0 (0%)	11 (48%)
NA	2	1
Histology, *n* (%)		
Urothelial carcinoma	16 (80%)	23 (96%)
Squamous cell carcinoma	1 (5%)	0 (0%)
Neuroendocrine carcinoma	2 (10%)	0 (0%)
Adenocarcinoma	1 (5%)	1 (4%)

a No evidence of disease.

b TUR-BT, includes intra- and extra-vesical recurrences and T-class progressions; RC, includes local residual diseases and distant metastases.

c Lost to follow-up after the disease recurred.

### Ethics

The study was approved by the Ethics Committee of the Hospital District of Southwestern Finland (reference: 3/1801/2013). The samples were collected in collaboration with the Auria Biobank (Turku, Finland) with the approval of the Scientific Steering Committee of Auria (reference: TO3/021/18). All patients signed an informed consent form to participate in this study.

### Sample collection

Fresh tissue specimens were obtained from TUR-BT or RC. After RC, study specimens were collected from the tumours and macroscopic benign-looking areas of the bladder mucosa. One half of each tissue specimen was snap-frozen in liquid nitrogen and stored at −80 °C, whereas the other half was fixed in formalin for 24 h and embedded in paraffin following standard protocols for sample validation. The collected TUR-BT tumour specimens were placed in PBS at RT, minced into smaller pieces, snap-frozen in liquid nitrogen, and stored at −80 °C.

Blood samples were collected under spinal or general anaesthesia at the beginning of TUR-BT or RC. Samples collected in serum tubes were stored at RT for 30 min before being centrifuged (2300 × G, 12 min) and stored at −80 °C.

Frozen samples were locally reviewed by an experienced uropathologist (PT) who microscopically examined the adjacent formalin-fixed paraffin-embedded (FFPE) tissue specimens. Tumour samples with a minimum of 15% tumour cells were considered representative. The research data were merged with clinical data from a prospectively maintained database.

### Mass spectrometry analyses

As previously described, LC-MS/MS analysis was used to determine the levels of 23 steroids in freshly frozen tissue and serum samples.^26^ Briefly, freshly frozen tissue samples were homogenised in cold saline (ratio of 50 mg of tissue in 500 μl saline; 9 mg/ml, B. Braun Medical, Finland) using an Ultra Turrax T25 (IKA Werke, Germany) and Qiagen TissueLyser LT (RRID: SCR_020428) on wet ice and then stored at −80 °C until use.

Steroids were extracted using toluene from a 150 μl tissue homogenate or serum sample, spiked with isotope-labelled steroids as internal standards. Hydroxylamine was used to improve the ionisation. LC-MS/MS analysis was performed using Agilent 1290 UHPLC and Agilent 6495 QQQ. Steroid concentrations of RC tissue, TUR-BT tumour, and serum samples were measured in separate runs.

The calibration curve was refitted to estimate the serum testosterone levels above the normal quantitation range. The amount of DHT was measured using two different isomers in RC and serum samples. In both cases, a second measurement was performed if the first measurement was outside the calibration range. The amounts of steroids were normalised to pmol/g of the original tissue samples by multiplying the measurements by the volume of the tissue homogenate and dividing by the mass of the original tissue sample. Steroid levels below the lower limit of quantitation were considered zero in the analysis (Supplementary Materials). One gram of the tissue sample was considered equivalent to 1 ml of serum.

### RNA extraction and sequencing

Freshly frozen tissue samples were homogenised using an Ultra Turrax T25 (IKA Werke, Germany), and RNA was extracted using an AllPrep DNA/RNA Mini kit (Cat# 80204, Qiagen, CA, USA) according to the manufacturer's protocol. Next-generation sequencing (NGS) was performed at the Finnish Functional Genomics Center (Turku, Finland). RNA quality was determined using an Advanced Analytical Fragment Analyzer (Agilent, Santa Clara, CA, USA), and the RNA concentration was measured using a Qubit Fluorometer (Life Technologies).

The library was prepared from 100 ng of RNA using the TruSeq Stranded mRNA HT kit (Illumina). Library quality was ensured using an Agilent Bioanalyzer and Fragment Analyzer, and the concentration was measured using a Qubit Fluorometric Quantitation.

RNA sequencing was performed using an Illumina NovaSeq 6000 (RRID:SCR_016387) (a read length of 2 × 100 bp). More than 85% of bases higher than Q30 were expected. Data were extracted using standard bcl2fastq2 conversion software (RRID:SCR_015058).

### RNA data processing and expression analysis

The quality of the raw sequencing reads was analysed using FastQC (version 0.11.8, RRID:SCR_014583). The reads were aligned using the R/Bioconductor (RRID:SCR_006442) package Rsubread (version 2.6.4, RRID:SCR_016945) to the hg38 reference genome derived from Illumina iGenomes (https://support.illumina.com/sequencing/sequencing software/igenome.html (UCSC)) and gene-wise read counts were produced based on the RefSeq gene models. The read counts were normalised using edgeR (version 3.34.1, RRID:SCR_012802). Differential expression analysis (DGE) was performed using ROTS (version 1.20.0).^27^ Genes with count per million (CPM) expression values below one in more than half of the replicates in both the compared sample groups were filtered out prior to statistical testing. Absolute fold-change above 1.5 and false discovery below 0.01 were used as thresholds for the genes selected as differentially expressed. Volcano plots were generated using EnhancedVolcano (version 1.18.0, RRID:SCR_018931). Molecular classifications were performed using the MIBC consensus subtypes,^28^ and UROMOL2021 classes for NMIBC.^29^

### Immunohistochemistry

We constructed a tissue microarray (TMA) including three to four 1 mm punch biopsies from each tumour and non-tumour FFPE specimen. Immunohistochemistry was performed at the Department of Pathology, Turku University Hospital (Turku, Finland) using a BenchMark ULTRA IHC/ISH automated staining instrument (Ventana, Arizona, USA) according to the routine protocol. The slides were stained with a mouse monoclonal anti-Androgen Receptor antibody (Cat# NCL-AR-318, RRID:AB_442040, dilution 1:10, Leica Biosystems) and a rabbit monoclonal anti-Progesterone Receptor antibody (Cat# 790-4296 (also 790-2223), RRID:AB_2335976; Ventana) with positive and negative controls.

The stained slides were digitalised using a PANNORAMIC P1000 (3DHISTECH, Hungary) at 20-fold magnification and 40× resolution. The slides were converted from mrxs to mrxs using SlideConverter (v. 2.4.1, 3DHISTECH) before they were analysed using QuPath (v. 0.3.2; RRID:SCR_018257). DAB intensity was measured for each nucleus and classified semi-quantitatively as 0, negative; 1, weak; 2, moderate; and 3, strong.

The AR H-score was calculated for each TMA core by multiplying the nuclear staining intensity (0, 1, 2, and 3) by the corresponding percentage of positive tumour cells. Finally, the mean H-scores were used for further analyses.

### Statistical analysis

Statistical analysis was performed using R 3.6.1 and 4.3.1 (RRID:SCR_001905). All statistical analyses except survival analysis were preplanned. No power analysis was performed beforehand due to the limited availability of suitable patient sample material and the lack of published information about the bladder steroid environment needed for estimation. Therefore, the sample size was considered retrospectively based on the precision of the estimates. No null hypothesis significance testing was performed, and p-values are provided for legacy purposes only.

Tumours other than urothelial carcinoma were excluded from the statistical analysis. Steroid concentration was considered zero if the measured level was lower than the limit of quantitation.

Heatmaps were generated using pheatmap (version 1.0.12; RRID:SCR_016418). Steroids with less than two non-zero values were excluded. Sample-specific steroid values were further normalised by subtracting the mean and dividing it by the standard deviation across the samples. Tissue ratios were log_2_ transformed. The relationships between steroid levels in different sample groups were assessed by estimating the median of differences (paired samples) or difference in medians (non-paired samples) with 95% bias-corrected and accelerated bootstrap (10,000 bootstrap resamples with replacement) confidence intervals (95% CI). Estimations and 95% CIs were calculated using the confintr (v. 1.0.2; RRID:SCR_025098). Estimation plots were constructed using dabestr (version 0.3.9999; RRID:SCR_022340). To examine clustering patterns of steroid samples, principal component analysis was performed based on a correlation matrix using the stats package of R without a further rotation performed. The two largest components were used for 2D illustration, and steroids with less than six non-zero values were excluded. Spearman correlation analysis was used to analyse associations between tissue and serum steroid levels when their standard deviations were non-zero.

Post hoc survival analyses of patients included in the TURBT cohort were performed using the following R packages: survival version 3.5–5 (RRID:SCR_021137) and tidycmprsk version 0.2.0 (RRID:SCR_025053) and visualised using ggsurvfit version 0.3.0 (RRID:SCR_025045). HR was estimated using both a Fine–Gray sub-distribution hazard model and cause-specific hazard model as not all assumption for the latter were robustly met. Our primary event of interest was the first recurrence of the disease. The follow-up time was defined from the start time (the time of the operation, which also served as the origin for the analysis) to the occurrence of the event of interest, a competing event (cystectomy with pT0N0 status, or death due to another cause), loss to follow-up, or the last contact, whichever occurred first. Notably, none of the included patients died due to bladder cancer and thus it was not included as a competing risk. The proportional hazard assumption was evaluated by Schoenfeld residuals or testing interaction with time in the case of sub-distribution hazard model.

The median follow-up time of patients without events is reported. As routine follow-up cystoscopies are ceased after 5-year recurrence-free survival, longer follow-up periods were truncated to 60 months in the analysis. Four patients had MIBC and were excluded from the analysis because of non-curative surgery. The steroid-hot and steroid-cold groups were defined using unsupervised clusters for the serum steroid level-based analysis. For testosterone and *AR* expression analysis, tumours were stratified as low expression levels in both or high expression in either one based on histograms of testosterone concentration and *AR* expression. Two patients in the survival analysis experienced multiple events during the follow-up period: one patient with recurrence and one with later RC, who later died due to another cause. These or possible multiple recurrences are not included in the analysis. The low effective sample size precluded multivariable modelling.

### Role of funders

The funders had no role in study design; in the collection, analysis, and interpretation of data; in the writing of the report; or in the decision to submit the paper for publication.

## Results

To investigate the steroid profile of bladder cancer, we conducted simultaneous LC-MS/MS analysis of 23 steroids in tumour, serum, and adjacent non-tumour bladder tissue samples (Fig. 1a and b). Fresh frozen tissue and serum samples were used for all steroid measurements, as organic solvers drain steroids from FFPE specimens.^30^ The corresponding tissue samples were subjected to mRNA sequencing and histopathological validation. Regarding steroid measurements, 11α-OHA4 levels were undetectable in all samples and were therefore omitted. The batch effect of intratumoural steroid level measurements between the RC and TUR-BT cohorts, probably attributable to different sample collection methods, was considered too large for reliable comparison of distinct biological differences between the groups (Supplementary Materials). TUR-BT cohort's median follow-up period was 75.2 months (IQR = 71.4–83.5).

### Defining the general steroid profile of bladder cancer

**Fig. 2:**
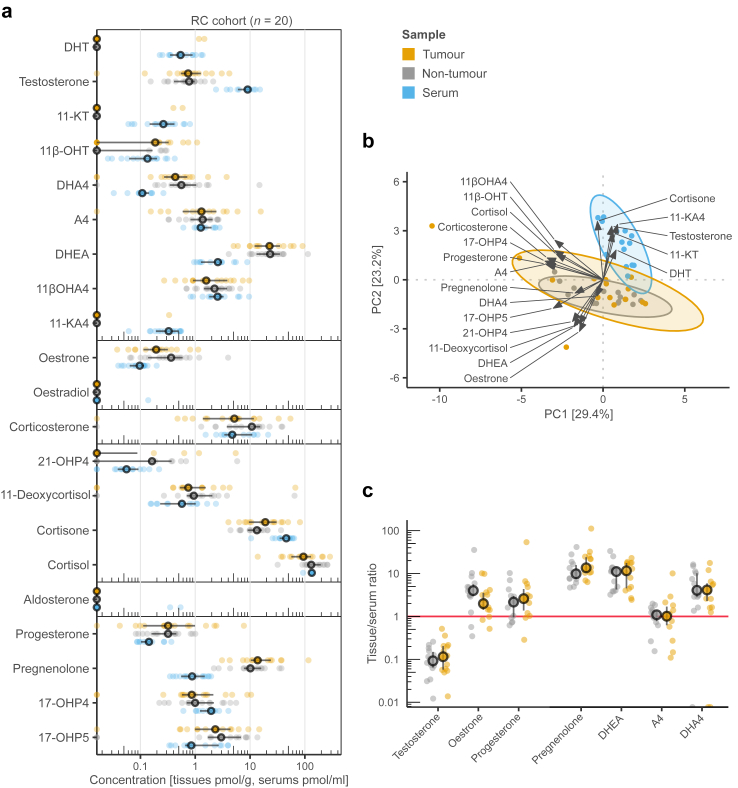
**Steroid profile of bladder cancer**. **a** Plot of steroid profiles in the RC cohort. **b** Biplot of principal component analysis (PCA) of 19 steroids in the tumour, adjacent non-tumour, and serum samples in the RC cohort (n = 16). Steroids with less than five unique concentration measurements were excluded. The arrows indicate the share of each steroid's contribution to PC1 and PC2 axis. **c** Plot showing tissue/serum ratios of testosterone (T), oestrone (E1), progesterone (P4), and precursor steroids pregnenolone (P5), DHEA, androstenedione (A4), and androstanedione (DHA4). Medians and IQRs are shown. One gram of tissue was considered equal to 1 ml of serum. Only urothelial carcinoma (UC) cases were included.

Steroid profiles are presented in Fig. 2a and Supplementary Table S1 for the RC cohort and in Supplementary Table S2 for the TUR-BT cohort. Notably, the most potent androgens (DHT and 11-KDHT) and highly potent oestradiol were unquantifiable in almost every tissue sample (including bladder adenocarcinoma). However, a squamous cell carcinoma tumour had DHT at a similar level (8.96 pmol/g) as previously reported for treatment-naïve primary prostatic adenocarcinoma (8.24 pmol/g),^31^ and two UC tumours had DHT at the same levels as recurrent prostate cancer during ADT (1.17 and 1.47 pmol/g vs 1.25 pmol/g).^32^ Furthermore, median serum levels of DHT (0.54 pmol/ml, IQR 0.35–0.89) and likely mostly testicular testosterone (9.0 pmol/ml, IQR 6.0–10) were found in physiologically low centiles (5–25%), as previously reported in elderly males.^33^^,^^34^

The principal component analysis (PCA) separated serum samples from tumour and non-tumour tissues in the RC cohort based on 19 steroids (Fig. 2b). Serum samples were dominated by potent androgens (e.g., DHT and testosterone), whereas oestrone and DHEA were present at low levels. We used tissue/serum ratios to investigate the relationships between serum and tissue steroid levels. Unsupervised clustering analysis of steroid tumour/serum ratios separated the basal and two stroma-rich clusters by molecular subtype in the RC cohort, whereas sparse histological subtypes were scattered (Supplementary Fig. S1a). In contrast, low- and high-grade tumours clustered together in the TUR-BT cohort (Supplementary Fig. S1b). The intratissue levels of potent androgens were only fractions of the corresponding serum levels (Fig. 2c and Supplementary Table S1), and there were no correlations between these variables in either cohort (Supplementary Figs. S2 and S3). Combined with the PCA results, these results suggest rapid androgen metabolism and, thus, a well-regulated androgenic environment in normal and cancerous bladder tissues.

In contrast to the potent sex steroids, the concentrations of the two precursor steroids, DHEA and pregnenolone, were 10–14-fold higher in the tissues than in the serum in the RC cohort (Fig. 2c, Supplementary Table S1). However, the median levels of androstenedione (the principal precursor of testosterone) appeared equal (tumours vs serum: difference of medians [95% CI] = −0.03 pmol/unit [−1.7, 0.50]; non-tumours vs serum: −0.11 pmol/unit [−0.62, 0.26]; P = 0.81, Friedman rank sum test). Moreover, the levels of precursor steroids were correlated between tumours and sera (Supplementary Fig. S2). In addition, we detected only minor RNA expression of steroid enzymes responsible for the *de novo* biosynthesis of the precursors (Supplementary Table S4). Thus, lipophilicity-driven cellular accumulation provides a plausible explanation for high intratissue concentrations of DHEA and pregnenolone.^35^

Of lifestyle factors, only patients’ smoking status was available. In the PCA analysis, steroid profiles were not clustered based on smoking status (Supplementary Fig. S4).

Finally, concerning the two females serving as controls, potent steroids were similarly below the quantification limit (e.g., DHT, oestradiol) or lower than in males (testosterone) in both tumours and sera, which is consistent with patients’ post-menopausal status (Supplementary Table S2).

### Comparisons of MIBC tumours and adjacent non-tumorous bladder tissues

**Fig. 3:**
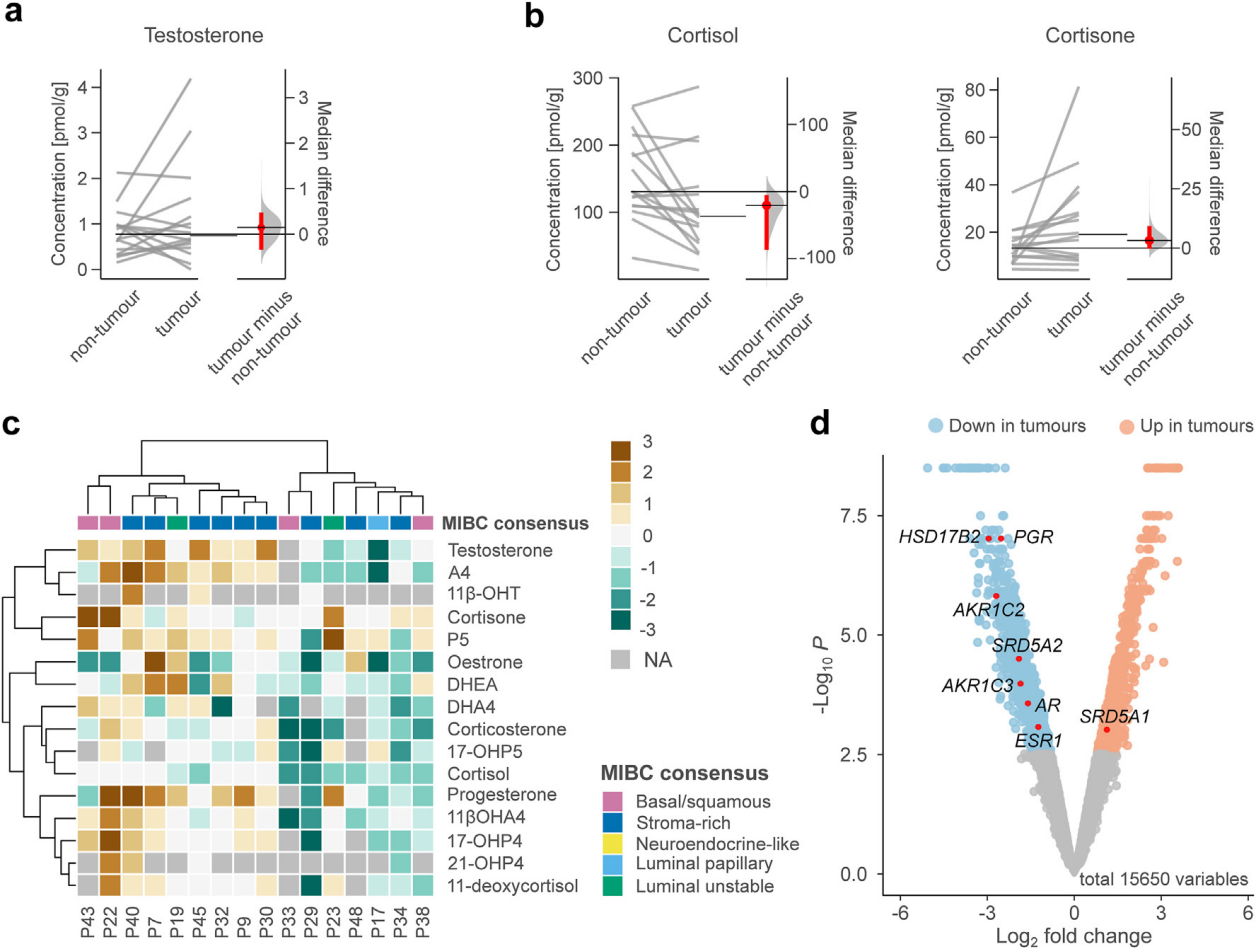
**Comparison between tumour and adjacent non-tumour samples**. Paired estimation plots for testosterone levels (**a**) and glucocorticoid concentrations (**b**) in paired tumour and non-tumour samples (n = 16). Both groups are plotted on the left axis with black lines indicating medians, and the paired median difference is plotted on the right axis. The median difference and 95% bootstrap CIs are depicted as red dots and lines, respectively. **c** Heatmap showing unsupervised clustering of intratissue steroid levels using the log_2_ of the tumour/non-tumour ratios. Only UC cases are included. **d** Volcano plot showing differentially expressed genes between tumour and non-tumour samples.

A comparison of the RC cohort's paired UC tumour and non-tumour samples revealed no difference in median testosterone levels (median of paired differences [95% CI] = 0.15 pmol/g, [−0.34, 0.47]; P = 0.39, Wilcoxon signed rank test; Fig. 3a, Supplementary Table S3). Cortisol levels appeared to be lower in the tumours (median of paired differences [95% CI] = −21 pmol/g [−87, −5.2)]; P = 0.021, Wilcoxon signed rank test), but cortisone levels were more similar (median of paired differences = 3.2 pmol/g [−0.34, 9.3]; P = 0.028, Fig. 3b). Unsupervised clustering analysis of the tumour/non-tumour steroid ratios segregated the tumours into groups with excess steroids (left) and the rest (right), irrespective of the molecular subtypes (Fig. 3c), suggesting that specific subtypes are not the principal drivers of intertissue steroid variation.

RNA sequencing revealed that enzymes that inactivate potent sex steroids (*HSD17B2* and *AKR1C2*) and progesterone (*AKR1C1*), as well as genes encoding steroid receptors (*AR*, *ESR1*, and *PGR*), were downregulated in MIBC tumours (Fig. 3d, Supplementary Table S4). In support of this, IHC analysis showed that PR was primarily expressed in benign stromal cells (Supplementary Fig. S5), which corresponded with a previous study.^36^ Regarding enzymes producing DHT, tumours expressed elevated levels of *SRD5A1*, whereas *SRD5A2* was mostly elevated in non-tumour samples, consistent with a previous study.^37^ Unsupervised clustering analysis of steroid receptors and metabolising enzymes similarly separated the tumour and non-tumour samples (Supplementary Fig. S6).

### Comparisons of high-to low-grade NMIBC tumours

**Fig. 4:**
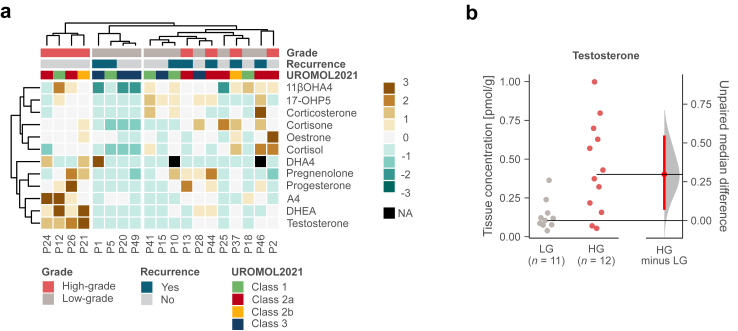
**Comparison of intratumoural steroid levels between high- and low-grade NMIBC UC tumours in TUR-BT cohort**. **a** Heatmaps showing unsupervised clustering of intratumoural steroid levels. Patients who were operated with non-curative-intent were excluded. **b** Estimation plot of testosterone levels in high-grade (HG) and low-grade (LG) NMIBC tumour samples. Data are presented on the left axis, and differences in medians with 95% bootstrap CI (red bar) on the right axis.

Next, we performed an unsupervised clustering analysis on the NMIBC samples of the TUR-BT cohort to evaluate possible intratumoural steroidogenic variations across different tumour grades. Three distinct tumour clusters were identified (Fig. 4a): high-grade tumours with high androgen levels (left), low-grade tumours with low overall steroid levels (middle), and a mixed group of both grades with low androgen levels (right). Considering intratumoural potent sex steroid levels, median testosterone levels were higher in high-grade tumours (0.40 pmol/g, IQR = 0.20–0.65) compared to low-grade tumours (0.10 pmol/g, IQR = 0.08–0.14), with a median difference of 0.30 pmol/g, 95% CI = 0.07–0.56; P = 0.013, Wilcoxon rank sum test (Fig. 4b, Supplementary Table S5).

**Fig. 5:**
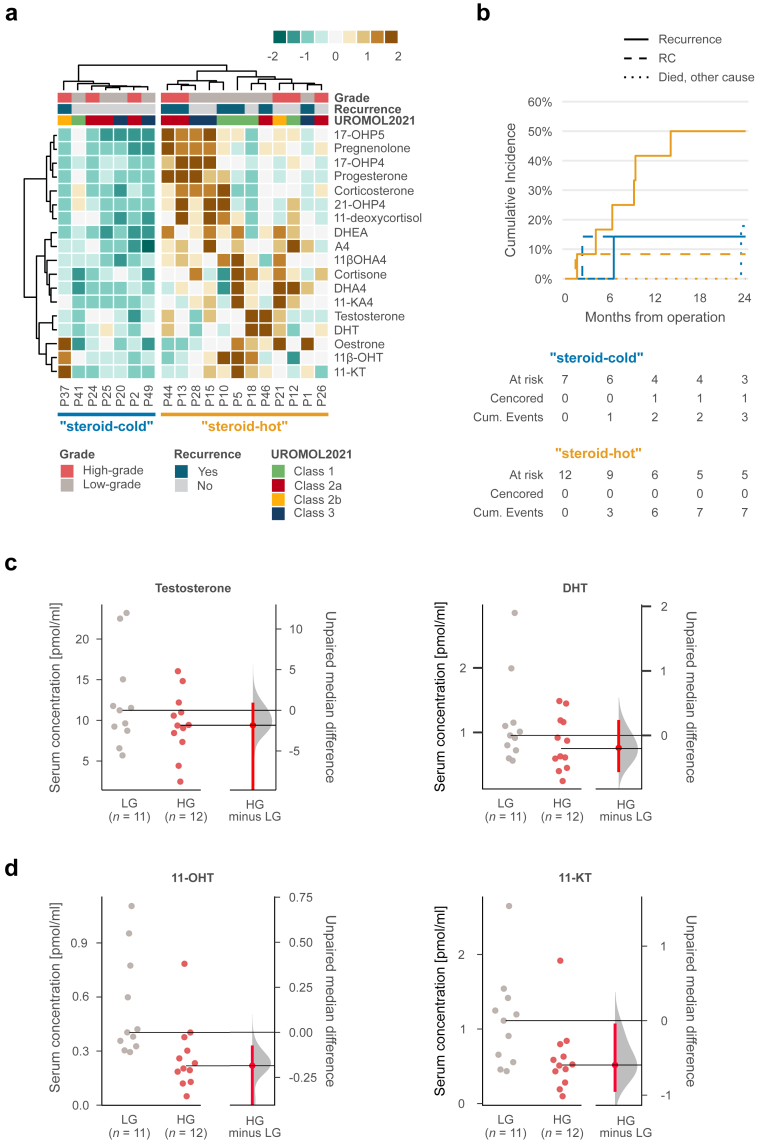
**Comparisons of serum steroid levels of patients with high- and low-grade NMIBC urothelial carcinoma tumours in TUR-BT cohort**. **a** Heatmap showing unsupervised clustering of serum steroid levels. Patients who were operated with non-curative-intent were excluded. **b** Cumulative incidence plots of steroid-hot and steroid-cold groups separated in Panel a. A plot with 60-month follow-up is shown in Supplementary Fig. S9. **c** Estimation plots of serum testosterone and DHT levels of patients with high-grade (HG) and low-grade (LG) tumours. **d** Estimation plots of serum 11-OHT and 11-KT levels of patients with high-grade (HG) and low-grade (LG) tumours. Data are presented on the left axis, and differences in medians with 95% bootstrap CI (red bar) on the right axis.

Regarding the serum steroid levels in the TUR-BT cohort, the overall low serum steroid levels separated a group of patients from the others (Fig. 5a), independent of tumour grade, in contrast to the tumour/serum ratios mentioned above (Supplementary Fig. S1b). Notably, seven (37%) out of 19 patients with NMIBC had recurrence during the follow-up: one (14%) in the steroid-cold group vs six (50%) in the steroid-hot group. The cause-specific hazard ratio was 3.76 (95% CI = 0.45–31.3; P = 0.2, log-rank test), and the sub-distribution hazard model produced a hazard ratio of 3.92 (95% CI = 0.51–30.2; P = 0.2, Gray's test; Fig. 5b).

Median differences [95% CI] in serum levels of potent androgens appeared to be minor (testosterone: −1.8 pmol/ml [−12, 0.94], P = 0.34; DHT: −0.20 pmol/ml [−0.96, 0.24], P = 0.34, Wilcoxon rank sum test, Fig. 5c, Supplementary Table S5), consistent with previous results.^21^ However, patients with low-grade disease seemed to have higher serum levels of potent ketoandrogens (11-KT difference in median [95% CI] = −0.59 pmol/ml [−0.96, −0.04], P = 0.029 and 11β-OHT −0.19 pmol/ml [−0.59, −0.07], P = 0.0042; Wilcoxon rank sum test, Fig. 5d).

**Fig. 6:**
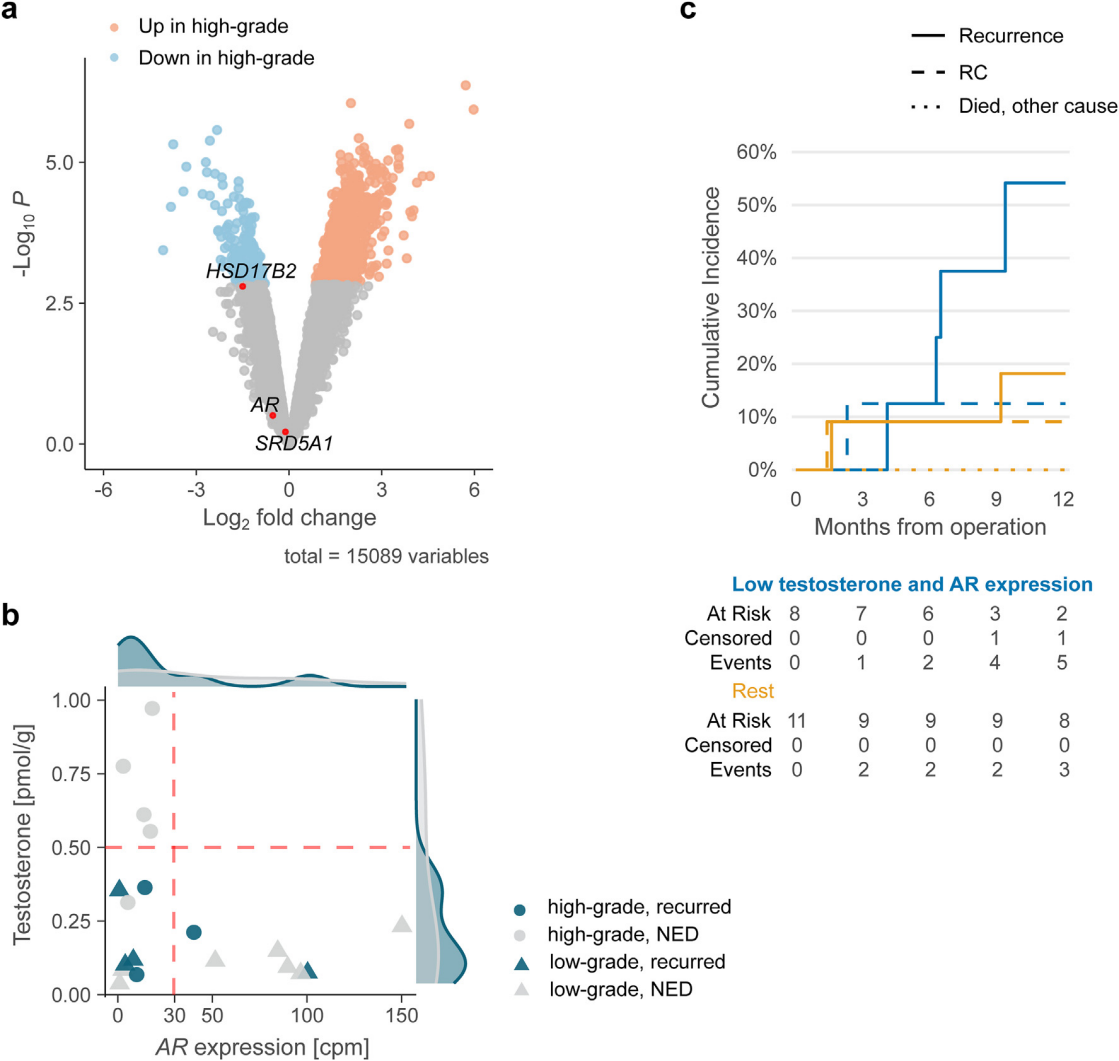
**The gene expression of steroid metabolising enzymes in high- and low-grade NMIBC tumours**. **a** Volcano plot of differential gene expression analysis between high- and low-grade tumours in the TUR-BT cohort. **b** Scatter plot showing the association between intratumoural testosterone levels and AR expression of NMIBC tumours. **c** Plot visualising the cumulative incidence of recurrence among tumours with low testosterone and *AR* expression (tumours in the bottom-left corner of the panel b) vs the rest. A plot with 60-month follow-up is shown in Supplementary Fig. S10. Only UC cases operated with curative intent are included. NED, No evidence of disease.

We did not observe any differences exceeding our filtering limits in the DGE analysis of the TUR-BT cohort (Fig. 6a, Supplementary Table S6). However, *HSD17B2* (inactivating potent androgens) expression was notably higher in low-grade tumours, suggesting impaired androgen inactivation in some high-grade tumours or increased metabolism in low-grade NMIBC tumours, potentially explaining the observed higher intratumoural testosterone levels.

Finally, we evaluated intratumoural testosterone levels in the TUR-BT cohort and compared them to *AR* expression (Fig. 6b). In contrast to serum androgen levels, patients with high intratumoural testosterone levels or *AR* expression had fewer recurrences (two out of 11 patients) than those with low levels (four out of eight patients). The cause-specific HR was 0.19 (95% CI = 0.04–1.01; P = 0.038; log-rank test) and sub-distribution HR was 0.29 (95% CI = 0.05–1.56; P = 0.2, Gray's test; Fig. 6c). In consideration of other recurrence-related factors, both patients with post-operative BCG-treatment and with low intratumoural testosterone levels experienced recurrence during the follow-up, whereas all three patients without BCG instillations but with high testosterone levels did not. Finally, we validated *AR* expression using IHC and rerun the analysis. Here, spearman correlation coefficient was 0.90 (95% CI = 0.74–0.96; P = <0.0001) and cumulative incidence functions resembled visually RNA level analysis, but hazard ratios were not calculated due to unmet proportional hazard assumption (Supplementary Fig. S7). Regarding high AR expression, these results correspond to a previously observed beneficial association between AR expression and recurrence-free-survival in bladder cancer.^11^^,^^37^

### Comparisons of serum levels between MIBC and NMIBC

As neither cohort contained enough MIBC and NMIBC tumours for formal analysis of steroid levels, we compared the serum steroid levels between MIBC and NMIBC using samples from both cohorts. The median serum levels of adrenal steroids appeared lower in MIBC cases (e.g., P5: difference in median [95% CI] = −1.2 pmol/ml [−2.5, −0.09]; P = 0.0030 Wilcoxon rank sum test; DHEA: −8.1 pmol/ml [−11, −3.6]; P = 0.0010), whereas the levels of gonadal and peripherally produced steroids (including potent androgens) were similar (e.g., testosterone difference in medians [95% CI] = −0.77 pmol/ml [−3.6, −1.5]; P = 0.080, Wilcoxon rank sum test; Supplementary Table S7). However, in the PCA analysis, MIBC and most recurrence-free NMIBC cases clustered together and were notably characterised by low levels of androgens (Supplementary Fig. S8).

### The expression of steroid metabolising enzymes across bladder tumours

**Fig. 7:**
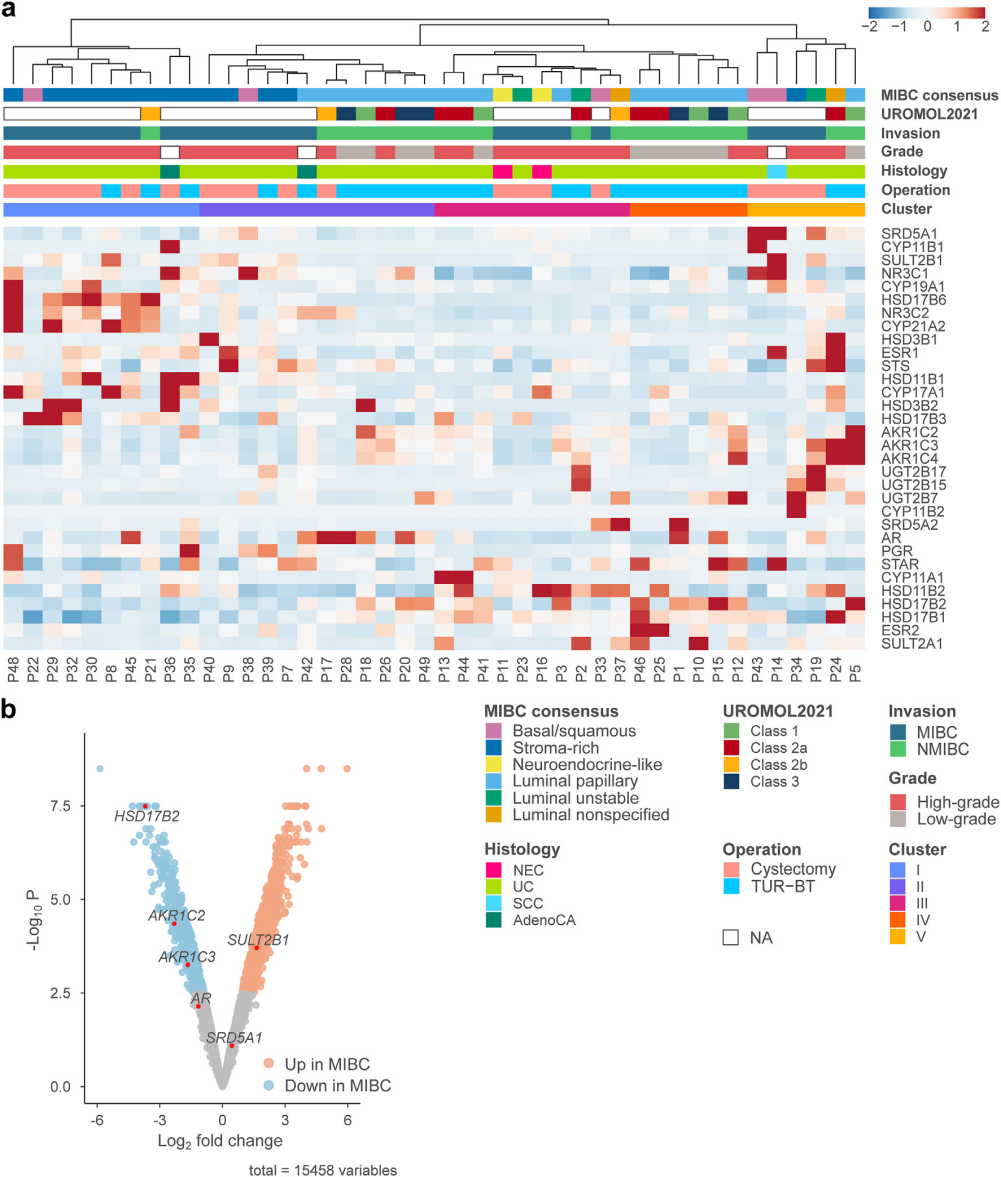
**The expression of steroid hormone-related genes in bladder cancer tumours. a** Heatmap showing gene expression of steroid metabolising enzymes and steroid receptors in tumour tissue samples from RC and TUR-BT cohorts. **b** Volcano plot of differential gene expression analysis between the tumour samples. AdenoCA, adenocarcinoma; NEC, neuroendocrine carcinoma; SCC, squamous cell carcinoma; UC, urothelial carcinoma.

To evaluate steroid metabolism in more detail, we analysed the expression of steroid-metabolising enzymes in the tumours of both cohorts. Unsupervised clustering analysis of the enzymes’ mRNA levels revealed several clusters that were associated with invasion status, grade, and MIBC consensus subtype of the tumours: muscle-invasive stroma-rich tumours (I); a cluster of luminal NMIBC and stroma-rich MIBC tumours that were further sub-clustered in a group including stroma-rich MIBC and luminal low-grade tumours (II), luminal high-grade tumours (III), luminal low-grade tumours (IV); and a mixed group of tumours with high expression of enzymes activating potent steroids (V) (Fig. 7a). Notably, in contrast to the MIBC consensus classification, the molecular subtypes of NMIBC tumours were not distinguished as clustering drivers. These results indicate significant steroidogenic heterogeneity among bladder cancer tumours.

Regarding the intratumor expression of androgen-inactivating enzymes, *HSD17B2* and *AKR1C2*, as well as the androgen activator *AKR1C3*, were downregulated in MIBC tumours compared to NMIBC tumours (Fig. 7b, Supplementary Table S8). These findings strongly suggest that the regulation of androgen metabolism is impaired as the stage of bladder cancer advances.

## Discussion

**Fig. 8:**
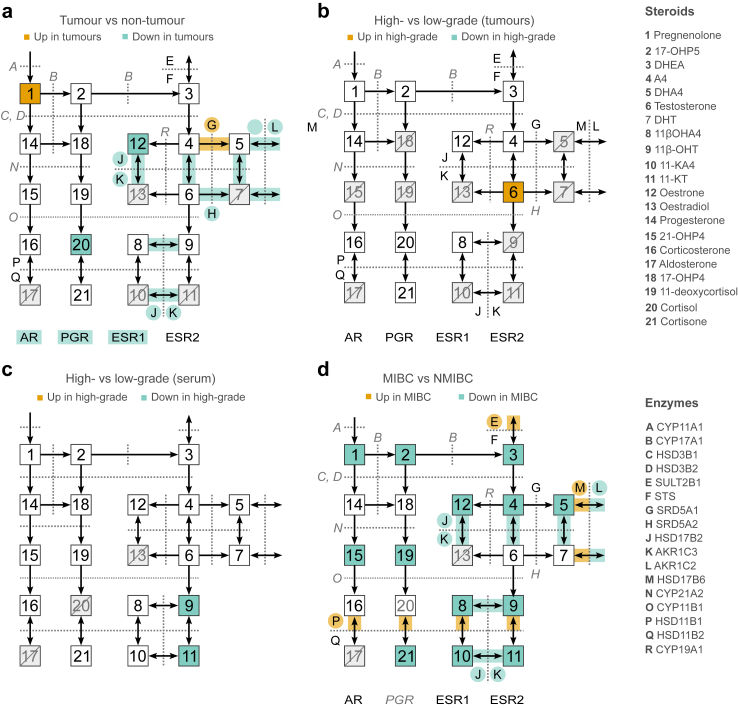
**Summarising steroid biosynthesis charts**. **a** Comparisons between tumour and non-tumour samples in the RC cohort. **b** Comparisons between high-grade and low-grade tumour samples in TUR-BT cohort. **c** Comparisons of steroid serum levels between high- and low-grade cases in the TUR-BT cohort. **d** Comparisons of serum steroid levels and intratumoural steroidogenic enzyme expressions between MIBC and NMIBC cases in both cohorts. Numbers indicate different steroids, and the fill colours of the boxes show the subgroups with considerably higher median steroid levels. Crossed-out boxes indicate insufficient unique measurements (n < 6). Letters indicate steroid enzyme and receptor genes, and the highlight colour indicates the subgroups with over 1.5-fold change and FDR under 0.01. Enzymes in grey and italics were filtered out from the analysis because of their very low expression levels.

Previous studies have associated AR expression and androgen signalling with bladder cancer,^3^^,^^7^, ^8^, ^9^^,^^11^^,^^15^^,^^38^ yet the intratumoural androgenic environment and the levels of utilisable ligands for AR have been unknown. In the current study, we conducted detailed LC-MS/MS and transcriptomic analyses to explore the intratumoural sex steroid milieu in bladder cancer. To the best of our knowledge, this is the most comprehensive steroidomics study to cover a panel of active sex steroids and their precursors to exploit both tissue and serum samples from patients with bladder cancer. We further compared the differences in steroid profiles between tumour and non-tumour tissues and between different grades and stages of the disease. The results are summarised in steroid biosynthesis charts for a comprehensive view (Fig. 8a–d).

Overall, we found that both tumour and non-tumour tissues were androgen-poor compared to patients’ serum levels, implying that circulating androgens are rapidly metabolised in both tumour and normal bladder tissues. In comparison with treatment-naïve primary prostatic adenocarcinoma,^31^ the median testosterone levels were 8-fold higher, whereas DHT and 11-KDHT were virtually unquantifiable in bladder cancer tumour samples, which portrays an essentially different androgen metabolism between these two malignancies.

Based on our results, the identified differences in androgen levels between bladder and steroid-depended cancers might be due to decreased activation of potent androgens from their precursor steroids (i.e., low *AKR1C3/4* expression levels). However, we also found the downregulation of enzymes that inactivate potent sex steroids, resembling the course of illness of classical steroid-dependent cancers.^39^ Notably, *HSD17B2*, which inactivates DHT and testosterone, was gradually downregulated during disease progression. A similar downregulation has been reported in public bladder cancer datasets.^40^ Furthermore, tumours expressed more *SRD5A1* than non-tumour samples, whereas *SRD5A2* expression was downregulated in the tumour samples. These results correspond to similar observations in the bladder and advanced forms of prostate cancer.^9^^,^^41^^,^^42^

Thus, as aggressive bladder cancer develops and grows in this castration-like environment, and the steroidogenic enzymatic milieu resembles castration-resistant prostate cancer, the direct effects of decreasing androgen levels in already arisen cancer cell clones might be limited. However, in our cohort, patients with NMIBC and low circulating steroid hormone levels had fewer recurrences than those with high steroid levels (Fig. 5b). This resembles the preclinical results that castrated male mice develop fewer chemically induced tumours.^7^ Although the local restraining effect of 5-ARIs on carcinogenesis may be negligible,^43^ possibly due to the above-observed lack of DHT production in the bladder, this result supports the previously proposed notion that reducing circulating DHT levels may benefit some patients, yet the results have been varying.^24^^,^^25^

In contrast to a higher recurrence rate of patients with high serum steroid levels, patients with NMIBC and relatively high intratumoural testosterone levels or high *AR* expression had a lower recurrence rate than those with *AR*-negative tumours and low testosterone levels (Fig. 6c). This is in line with previous studies, where intratumoural upregulation of *AR* has been associated with improved recurrence-free survival.^11^^,^^37^ We hypothesise that tumours with high intratumoural testosterone levels may comprise a subpopulation of tumours together with AR-positive tumours. This subpopulation seems to induce increased *AR* expression in an androgen-poor environment and vice versa, suggesting a compensatory mechanism between them, as observed in prostate cancer models.^44^ While taking advantage of the functions of the androgen axis, tumours might still retain some of the restrictive regulatory properties of the axis in place, leading to less aggressive disease (e.g., more papillary growth pattern) or a more effective treatment response.

While associations of serum and intratumoural steroid levels on the recurrence might seem contradictory at first glance, they represent different aspects of the disease. TUR-BT operation aims to remove any macroscopic tumour, followed by intravesical chemotherapy instillation(s) to eliminate any scattered tumour cells. Thus, the recurrence is caused by either *de novo* tumorigenesis or proliferation of scattered tumour cells resistant to the chemotherapy agents instilled postoperatively into the bladder. Serum steroid levels reflect systemic conditions in which circulating androgens may potentially stimulate the post-operative *de novo* tumorigenesis or proliferation of these scattered tumour cells. On the other hand, intratumoural steroid levels reflect more the intrinsic characteristics of tumour cells, and the tumours with intact androgen regulation and exophytic/papillary features become more likely removed entirely or may be more sensitive to chemotherapy instillation.^15^^,^^28^

The cause-specific HR and the sub-distribution HR were similar in the analysis of the association between serum steroid levels and recurrence, indicating that competing risks have minimal impact on the estimated risk of recurrence. In contrast, for intratumoural testosterone and *AR* expression levels, a more pronounced difference was observed: the cause-specific analysis suggested a lower risk of recurrence for patients with high testosterone or *AR* levels, while the sub-distribution analysis reflected similar, but slightly diminished protective effect when accounting for competing risks, such as cystectomy without residual tumour. This suggests that competing risks may have a more pronounced effect on the estimated risk in the latter case, however, the confidence intervals were wide and overlapping. Some degree of difference would be expected as our univariate model did not include common risk factors of recurrency and competing events, e.g., high tumour grade or aggressive BC subtypes.

In addition to androgens, the effects of oestrogen on bladder cancer have remained unclear. In our analysis, in line with the potent androgen results, highly potent oestradiol was unquantifiable in all the tissue samples. Weakly potent oestrone levels were lower in MIBC tumours than adjacent non-tumour samples, whereas intratumoural or serum oestrone levels were similar between low- and high-grade NMIBC tumours. In addition, as *CYP19A1* (aromatase) was filtered out from various performed DGE analyses owing to its low expression levels, it seems unlikely that the differences above in oestrogen levels are aromatase-driven. Consistent with a previous study,^28^ *ESR1* was expressed more in basal/squamous tumours, whereas *ESR2* expression was higher in a few low-grade luminal tumours.

Regarding the serum samples, adrenal steroids diverged between MIBC and NMIBC. The median testosterone and DHT levels were at the hypogonadal levels, which agrees with the previous results.^45^ Similarly, tumour grade was not associated with serum testosterone levels, which is consistent with the results of a previous study.^21^ This divergence between MIBC and NMIBC is probably due to a combination of multiple disease-specific and confounding factors, including a higher proportion of frail patients among MIBC cases, preoperative nutrition and infusion conditions, general anaesthesia, and systemic stress due to radical surgery.

Although our study, together with available epidemiological data, suggests that there could be a subpopulation of bladder cancer patients who could benefit from manipulation of the androgen levels, e.g., through 5-ARIs or ADT with LhRH analogues or antagonists, more data and especially prospective randomised clinical trials are needed before commencing such treatment strategy in bladder cancer. Although these pharmacological agents have well-documented side effects (e.g., risk of gynecomastia, decreased libido, and erection dysfunction), the risk profile would be relatively favourable when compared to systemic chemotherapeutic agents.

While utilisation of steroid levels for prognostic purposes is not yet practical to the extent of our study, there is a transition to replace the steroid hormone immunoassays with high-throughput LC-MS/MS assays in clinical laboratories. Thus, utilisation of such assays and determining multiple analytes at the time is expected to be well-established in future clinical laboratories in hospital environments, which would make it possible to measure steroid profiles if their prognostic value is further proved.

Our study has limitations. First, our research design aimed to investigate as pristine bladder cancer steroid profiles as possible, minimising confounding factors such as NAC or BCG instillations. Consequently, patients included in the RC cohort were older and frailer than patients with bladder cancer on average, potentially limiting the generalizability of the results. Additionally, our analysed cohorts included only males; thus, our results are likely not directly extendable to females as most female patients with bladder cancer are post-menopausal and thus, levels of potent sex steroids are low. Moreover, it is possible to measure steroids only from untampered tissue samples, which excluded the use of small early-stage tumours that were entirely delivered for routine FFPE-based diagnostics. These factors also imposed constraints on the sample size, thus control of confounding factors, and the precision (e.g., wide confidence intervals) and extent of statistical analysis (e.g., multivariable regression). Our unrealistically large estimates of hazard ratios and confidence limits suggest exaggeration relative to any natural effect (i.e., scarce data bias); these are likely caused by a sparse number of patients with recurrence in the tested groups (e.g., steroid-cold, and tumours with high testosterone levels or androgen receptor). Additionally, not all assumptions of cause-specific model were robustly met,^46^ such as, a presence of unmeasured risk factor (tumour grade). However, the results were in line with the results of Fine–Gray model. Another limitation is the built-in selection bias of hazard ratios included in all HR estimations. Nevertheless, our primary findings are robust even within the present sample size. Second, the batch effect between the cohorts prevented direct comparisons of intratumoural steroid levels. The batch effect presumably results from differences in the tissue collection methods, such as saline draining steroids from samples following TUR-BT. In an ideal setting, intratumoural heterogeneity or spatial factors could be considered, but this would require an even more challenging sample collection design.

In summary, our study sheds light on the previously obscure steroid hormone milieu of bladder cancer. We demonstrated that androgens in cancerous and benign urothelium are present at castration levels, and above all, potent DHT is virtually unquantifiable. We also detected similar cancer-stage-associated changes in the expression of steroid-metabolising enzymes, particularly in those that inactivate potent androgens, as observed in advanced forms of classical steroid-dependent malignancies. As bladder cancer cells thrive in this kind of environment, the direct effects of 5-ARI and ADT against already-developed cancer cell clones seem limited. However, the detected differences in the systemic and intratumoural steroid milieu between non-recurring and more aggressive NMIBC tumours are biologically interesting, and if reproducible in a larger patient population, a prospective randomised trial to study the efficacy of ADT in BC secondary prevention is warranted.

## Contributors

KK: Conceptualisation, formal analysis, investigation, visualisation, data curation, writing–original draft, writing–review and editing. JM: Formal analysis, visualisation, data curation, writing–original draft. TL: investigation, data curation. AL: Formal analysis, supporting supervision. MRH: Investigation, writing–review and editing. SA: Resources. LLE: Supporting supervision. PJB: Conceptualisation, equal supervision, investigation, funding acquisition, writing–review and editing. MP: Conceptualisation, equal supervision, funding acquisition, writing–review and editing. PT: Conceptualization, equal supervision, investigation, project administration, funding acquisition, validation, writing–original draft, writing–review and editing.

All authors read and approved the final manuscript. KK, JM, AL, and MP verified the underlying data.

## Data sharing statement

The individual-level sensitive health data used in this study are not publicly available due to national and European regulations (GDPR). Researchers can apply for the data via the Auria Biobank (https://www.auria.fi/biopankki/en/tutkijoille). The processed non-sensitive data generated in this study are available upon reasonable request from the corresponding author.

## Declaration of interests

JM reports funding for PhD studies from the University of Turku Graduate School. Other authors declare no potential conflicts of interest.
