# Supplementary material for: Profiling steroid hormone landscape of bladder cancer reveals depletion of intratumoural androgens to castration levels: a cross-sectional study
Source: eBioMedicine. 2024 Sep 28;108:105359. doi: 10.1016/j.ebiom.2024.105359 (PMC11459583; doi:10.1016/j.ebiom.2024.105359)
Supplement: Supplement [file mmc1.docx]

Profiling steroid hormone landscape of bladder cancer reveals depletion of intratumoural androgens to castration levels: a cross-sectional study

Kimmo Kettunen, Julia Mathlin, Tarja Lamminen, Asta Laiho, Merja R. Häkkinen, Seppo Auriola, Laura L. Elo, Peter J. Boström, Matti Poutanen, Pekka Taimen

Supplementary Data

Contents

[Supplementary Figure. Q-Q plot 1](#_Toc173705020)

[PCA analysis 2](#_Toc173705021)

[PCA RC tumours smoking (Fig. S4) 2](#_Toc173705022)

[PCA RC serum smoking (Fig. S4) 2](#_Toc173705023)

[PCA TUR-BT tumours smoking (Fig S4) 3](#_Toc173705024)

[PCA TUR-BT serum smoking (Fig S4) 3](#_Toc173705025)

[PCA RC cohort (Fig 2) 4](#_Toc173705026)

[PCA all serum samples (Fig S8) 4](#_Toc173705027)

[Supplementary Table. The quantitation limits for LC-MS/MS analysis. 5](#_Toc173705028)


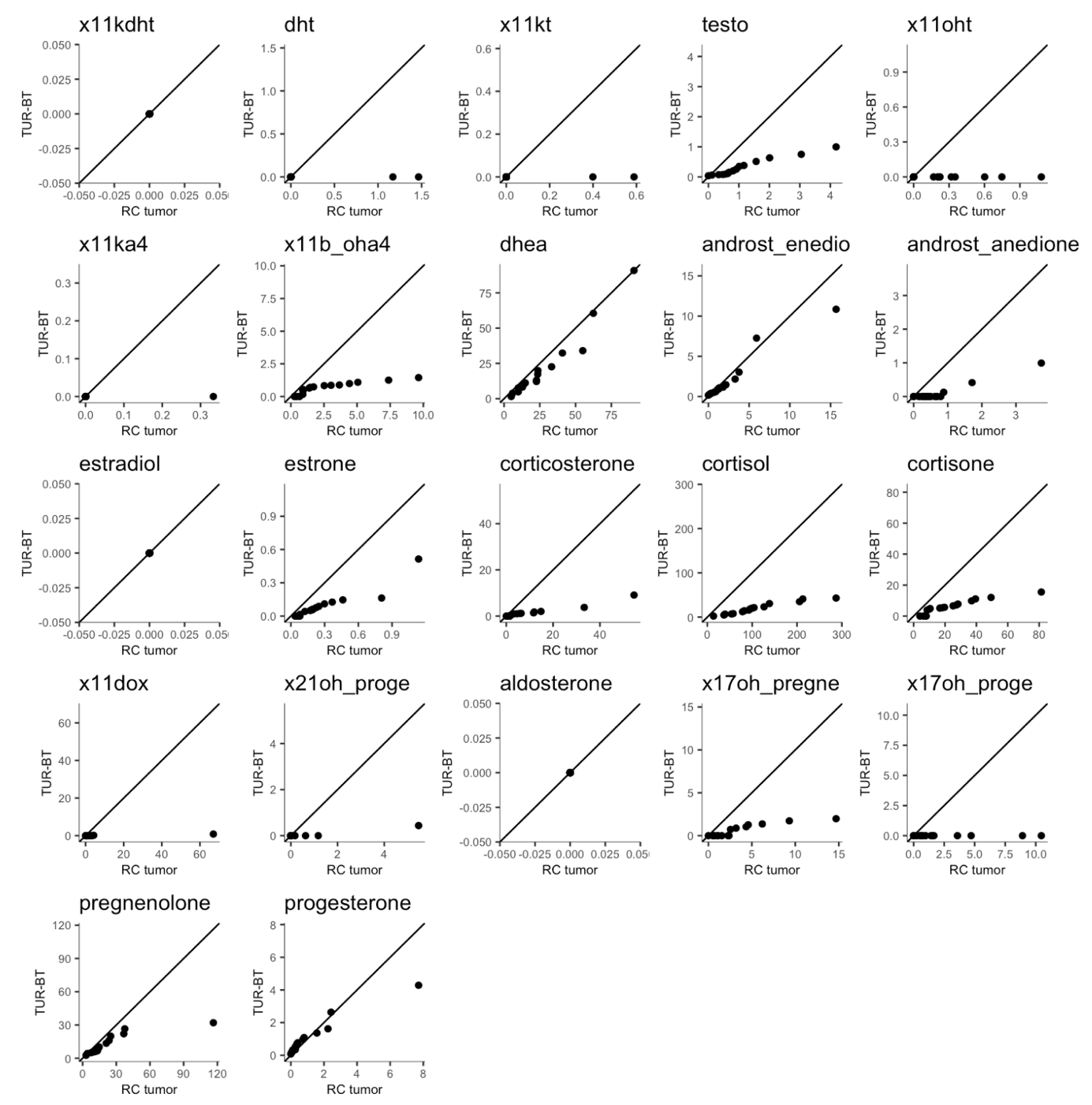


## Supplementary Figure. Q-Q plot

The Q-Q plots indicate large batch effect in **intratumoral steroid level** measurements **between the RC and the TUR-BT cohorts.**

## PCA analysis

### PCA RC tumours smoking (Fig. S4)


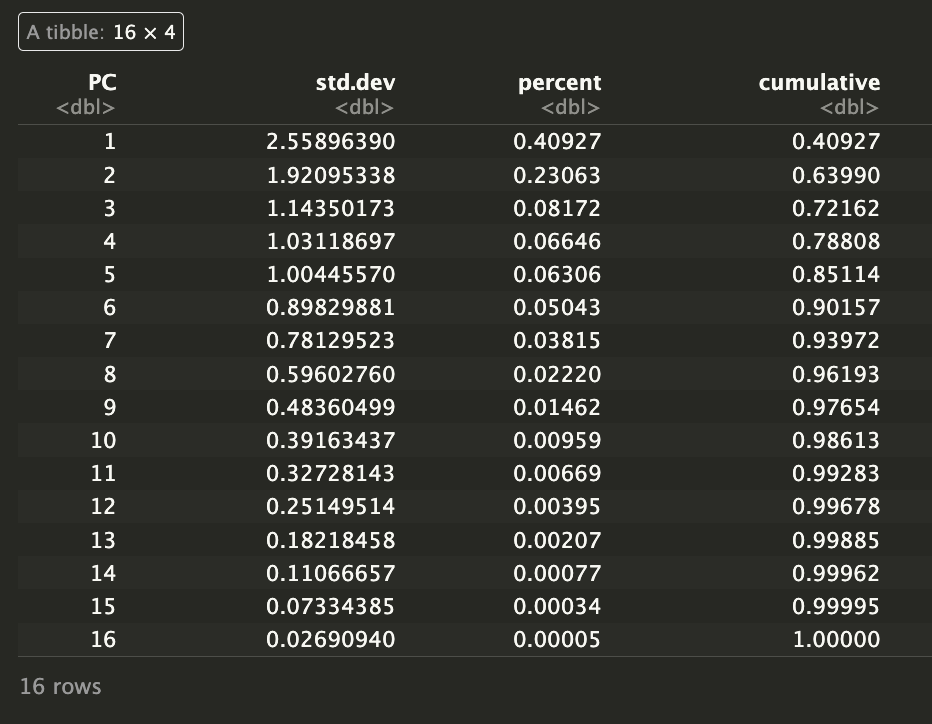


### PCA RC serum smoking (Fig. S4)


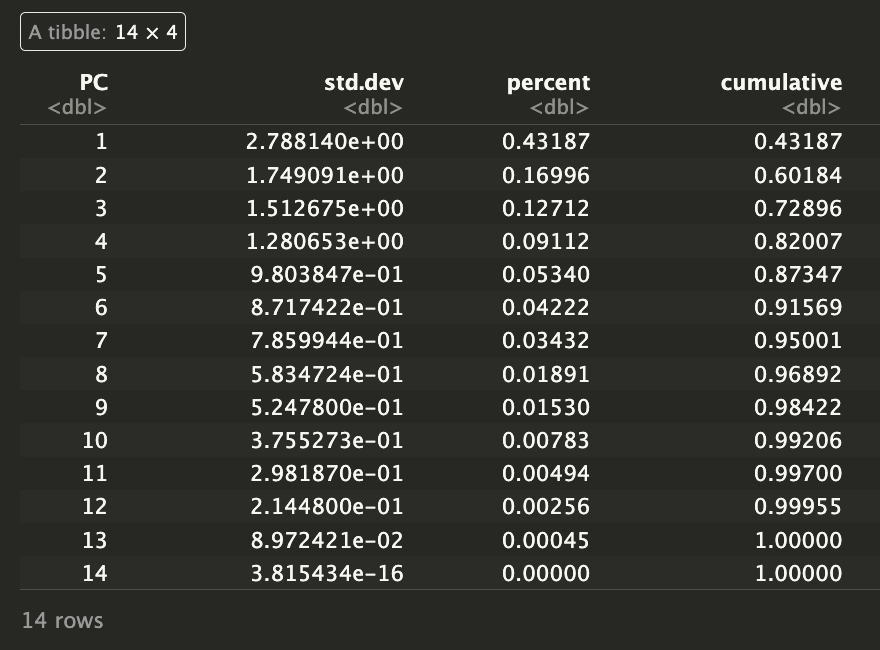


### PCA TUR-BT tumours smoking (Fig S4)


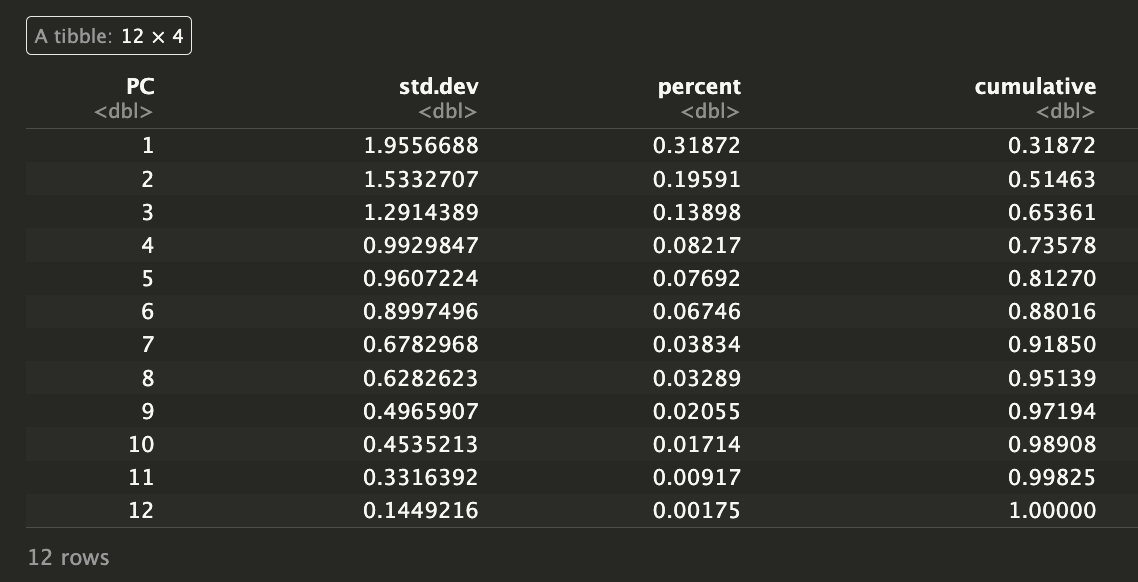


### PCA TUR-BT serum smoking (Fig S4)


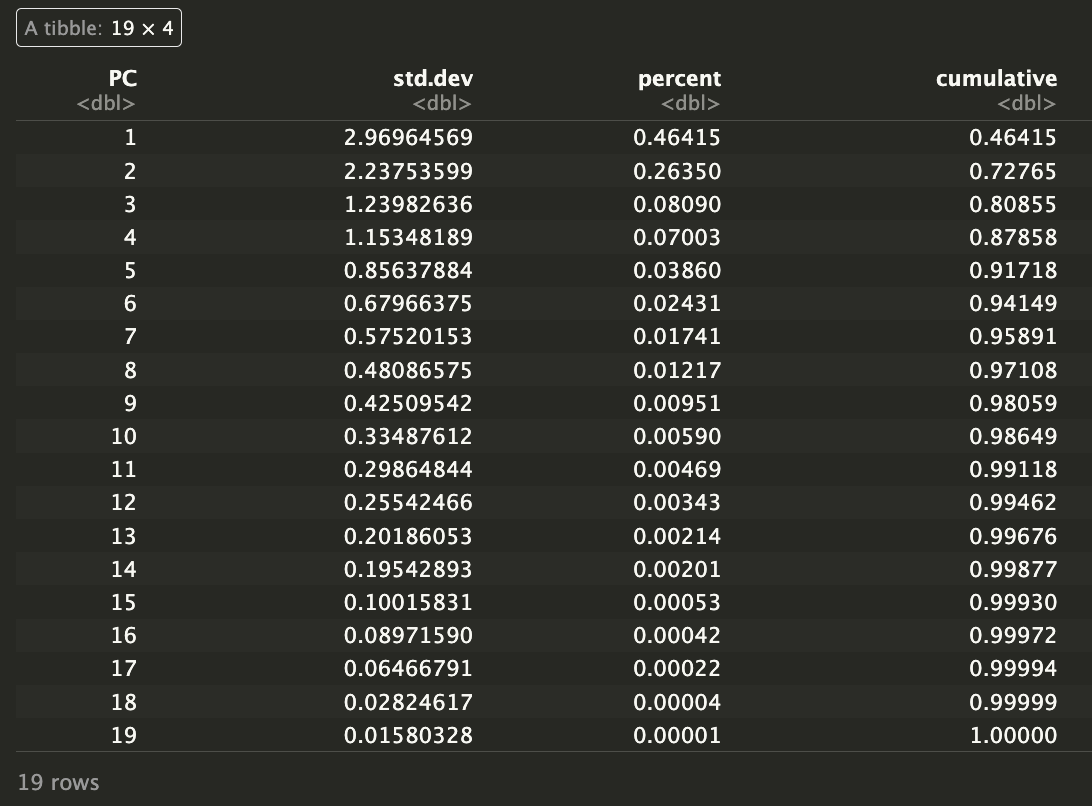


### PCA RC cohort (Fig 2)


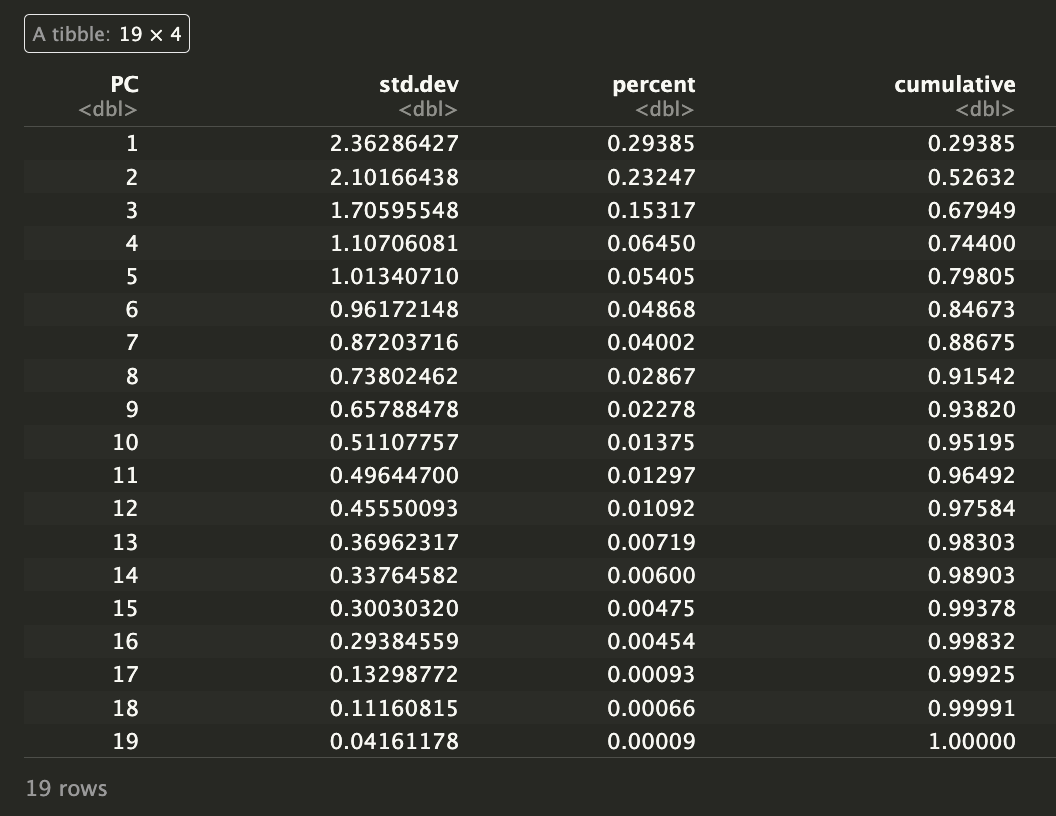


### PCA all serum samples (Fig S8)


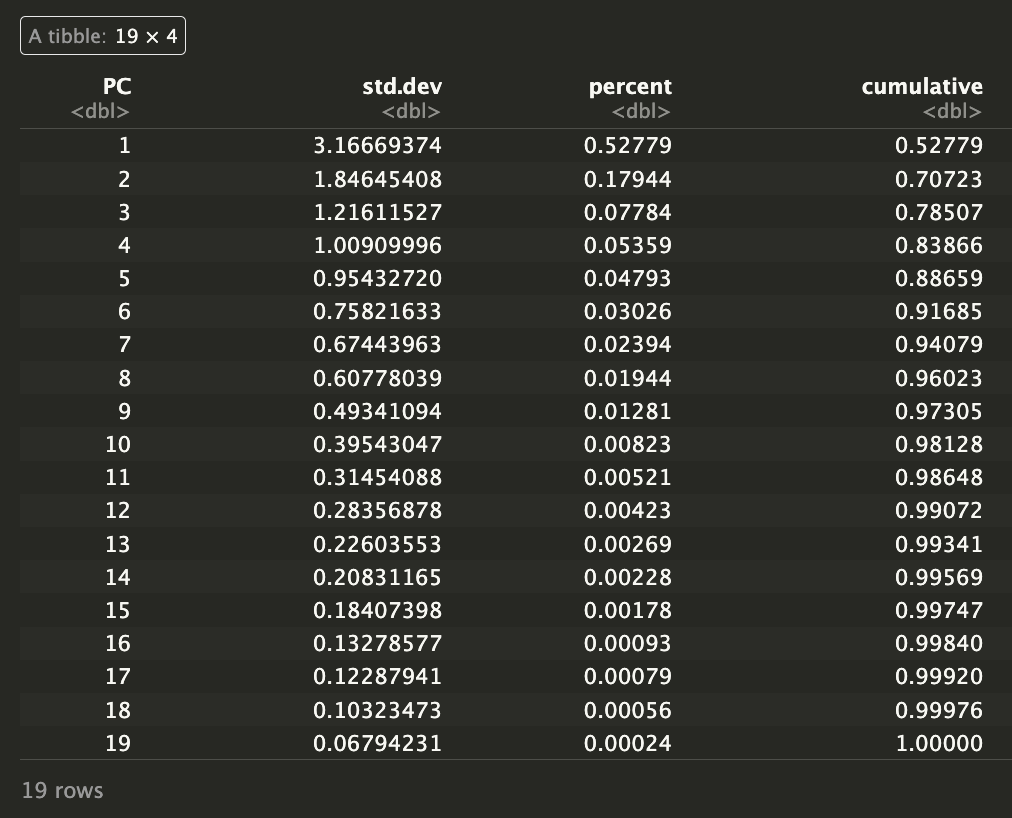


## Supplementary Table. The quantitation limits for LC-MS/MS analysis.

Three separate mass spectrometry runs (RC tissues, TUR-BT tissues, and all serum samples) used partially different calibration values for the lower limit of quantitation (lloq) and upper limit of quantitation (uloq). Units are pmol/l of serum or tissue lysate.

|  | **RC homogenates** | | **TUR-BT homogenates** | | **serum** | |
| --- | --- | --- | --- | --- | --- | --- |
| **Steroid** | **lloq [pmol/l]** | **uloq [pmol/l]** | **lloq [pmol/l]** | **uloq [pmol/l]** | **lloq [pmol/l]** | **uloq [pmol/l]** |
| 11⍺OHA4 | 13,2 | 13196 | 33 | 13196 | 13,2 | 13196 |
| 11βOHA4 | 13,4 | 13412 | 33,5 | 13412 | 33,5 | 33531 |
| S | 6,59 | 13184 | 33 | 13184 | 33 | 32959 |
| 11-KA4 | 33,1 | 13236 | 66 | 6618 | 33,1 | 13236 |
| 11-KDHT | 13,1 | 6574 | 33 | 6574 | 13,1 | 13148 |
| 11-KT | 33,3 | 6665 | 67 | 6665 | 13,3 | 13329 |
| 11β-OHT | 13,1 | 13133 | 33 | 13133 | 13,1 | 13133 |
| 17-OHPregnenolone | 31,8 | 12720 | 64 | 12720 | 31,8 | 31800 |
| 17-OHProgesterone | 13,3 | 13333 | 333 | 13333 | 66,7 | 13333 |
| 21-OHProgesterone | 12,9 | 12898 | 32,2 | 12898 | 32,2 | 12898 |
| Aldosterone | 128 | 12785 | 320 | 63924 | 320 | 63924 |
| Androstanedione | 13 | 12984 | 32 | 12984 | 13 | 12984 |
| Androstenedione | 6,64 | 13277 | 6,64 | 13277 | 33,2 | 33193 |
| Corticosterone | 33,4 | 13348 | 67 | 13348 | 33,4 | 66740 |
| Cortisol | 335 | 66950 | 67 | 13390 | 335 | 133900 |
| Cortisone | 131 | 65663 | 328 | 65663 | 328 | 131326 |
| DHEA | 33,3 | 33319 | 33,3 | 13328 | 33,3 | 33319 |
| DHT | 133 | 13320 | 33 | 13320 | 133 | 33299 |
| 2^nd^ isomer^1^ | 66,6 | 13320 |  |  | 66,6 | 33299 |
| Estradiol | 134 | 133960 | 335 | 133960 | 134 | 133960 |
| Estrone | 1,32 | 13177 | 3,29 | 13177 | 3,29 | 13177 |
| Pregnenolone | 32,9 | 13176 | 7 | 13176 | 32,9 | 13176 |
| Progesterone | 3,31 | 13259 | 6,6 | 13259 | 33,1 | 13259 |
| Testosterone | 3,32 | 13293 | 3,32 | 13293 | 33,2 | 13293 |
| 2^nd^ calibration^2^ |  |  |  |  | 332 | 33233 |
| ^1^Second isomer was measured to detect lower concentrations | | | | | | |
| ^2^Second calibration curve was fitted for serum samples to get more measurements | | | | | | |
