# Supplementary material for: Profiling steroid hormone landscape of bladder cancer reveals depletion of intratumoural androgens to castration levels: a cross-sectional study
Source: eBioMedicine. 2024 Sep 28;108:105359. doi: 10.1016/j.ebiom.2024.105359 (PMC11459583; doi:10.1016/j.ebiom.2024.105359)
Supplement: Supplementary Figures [file mmc2.docx]

Profiling steroid hormone landscape of bladder cancer reveals depletion of intratumoural androgens to castration levels: a cross-sectional study

Kimmo Kettunen, Julia Mathlin, Tarja Lamminen, Asta Laiho, Merja R. Häkkinen, Seppo Auriola, Laura L. Elo, Peter J. Boström, Matti Poutanen, Pekka Taimen

Supplementary Figures

Contents

[Supplementary Figure S1. 2](#_Toc1802163184)

[Supplementary Figure S2. 3](#_Toc1305120995)

[Supplementary Figure S3. 4](#_Toc114184395)

[Supplementary Figure S4 5](#_Toc213258183)

[Supplementary Figure S5 6](#_Toc1583879447)

[Supplementary Figure S6. 7](#_Toc770591466)

[Supplementary Figure S7. 8](#_Toc1728050318)

[Supplementary Figure S8. 9](#_Toc797391192)

[Supplementary Figure S9. 10](#_Toc1978410153)

[Supplementary Figure S10. 11](#_Toc295322208)

## Supplementary Figure S1.

Heatmap showing unsupervised log_2_ tissue/serum ratio clustering in the RC cohort (a) and the TUR-BT cohort (b). In the TUR-BT cohort, P42 had adenocarcinoma, and other patients had urothelial carcinoma. Row order is based similarly to unsupervised clustering.


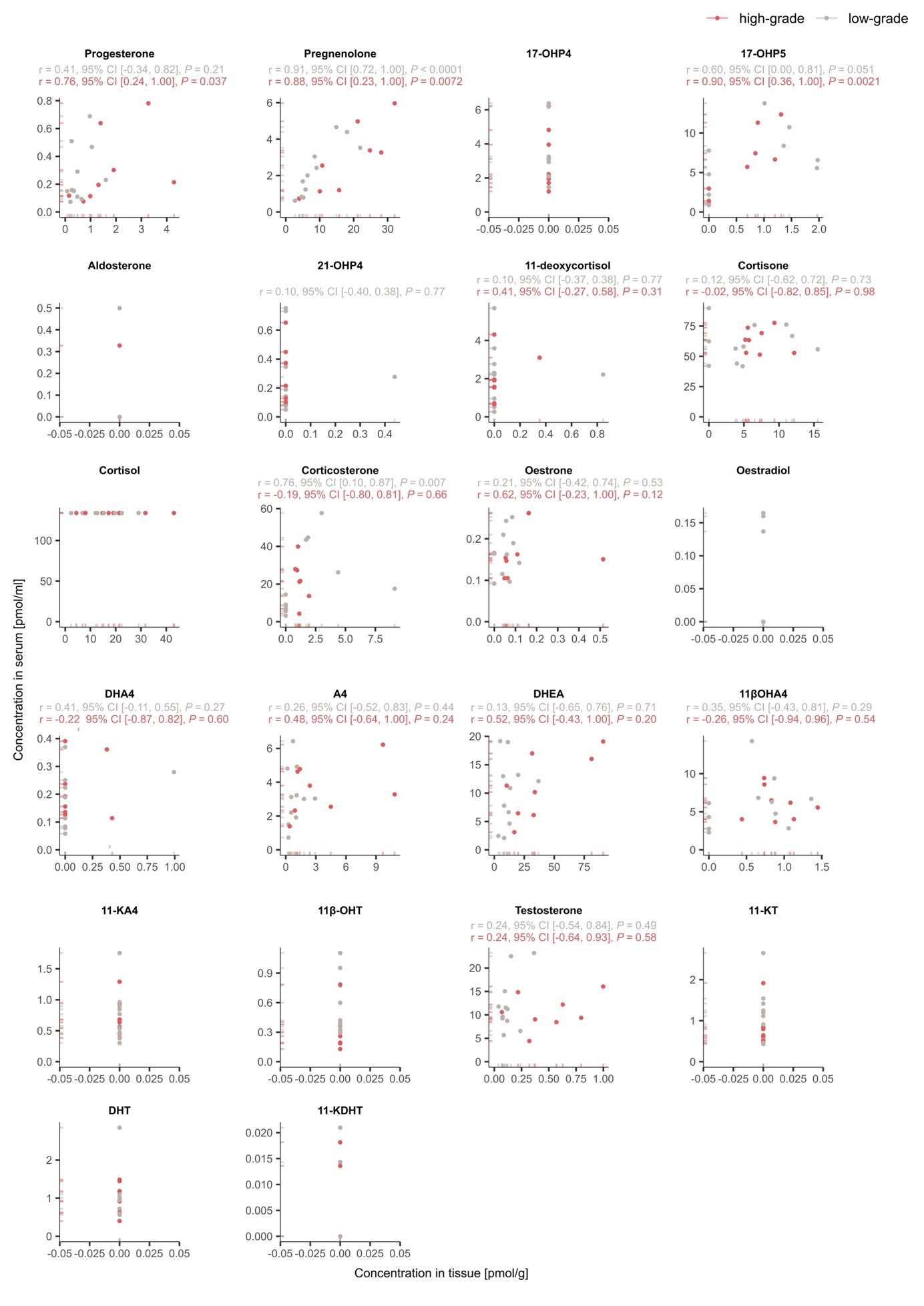


## Supplementary Figure S2.

Scatter plots showing Spearman correlations between tumour and serum samples in the TUR-BT cohort. P-values are not adjusted for multiple testing.


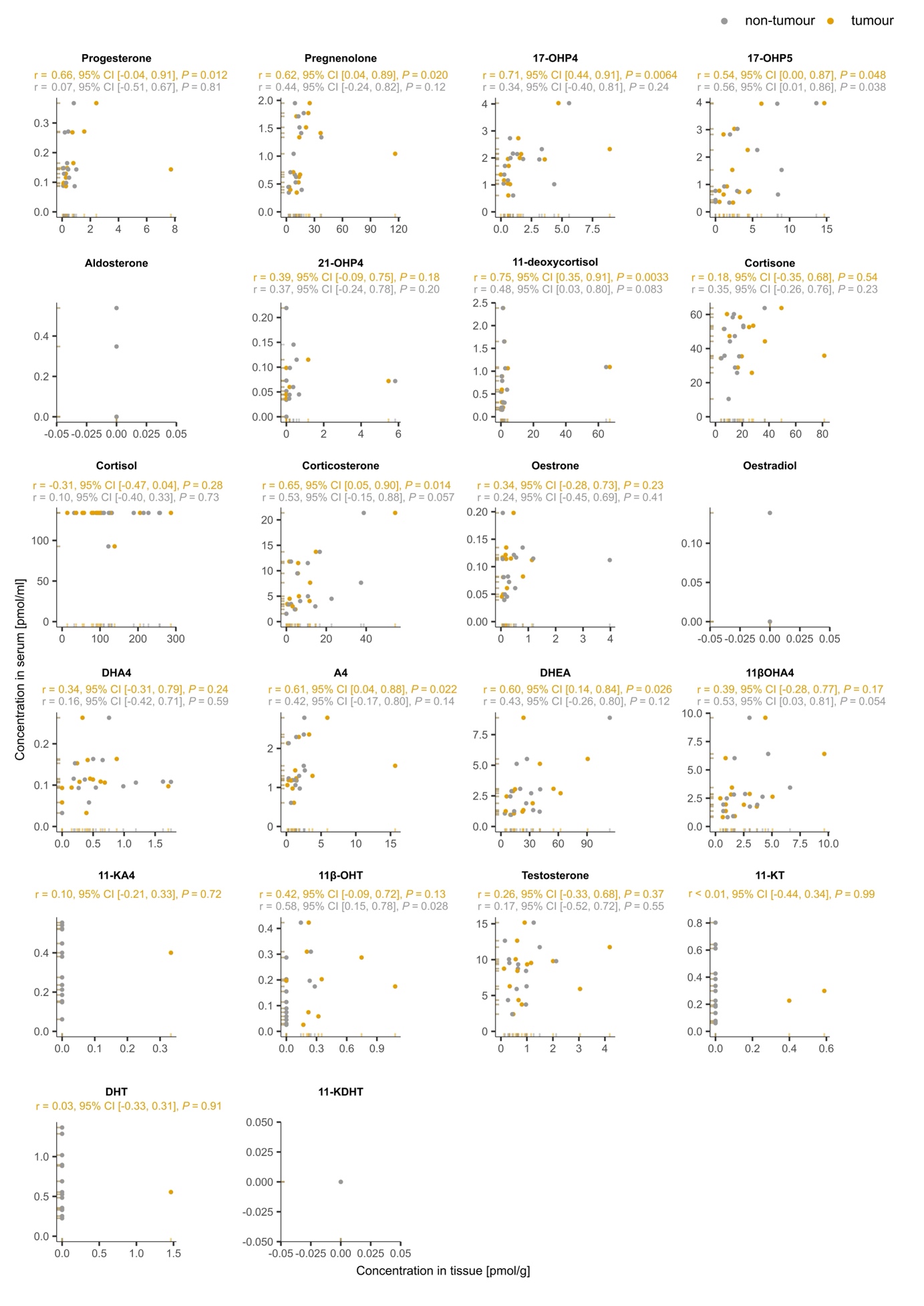


## Supplementary Figure S3.

Scatter plots showing Spearman correlations between tissue (tumour and non-tumour) and serum samples in the RC cohort. P-values are not adjusted for multiple testing.


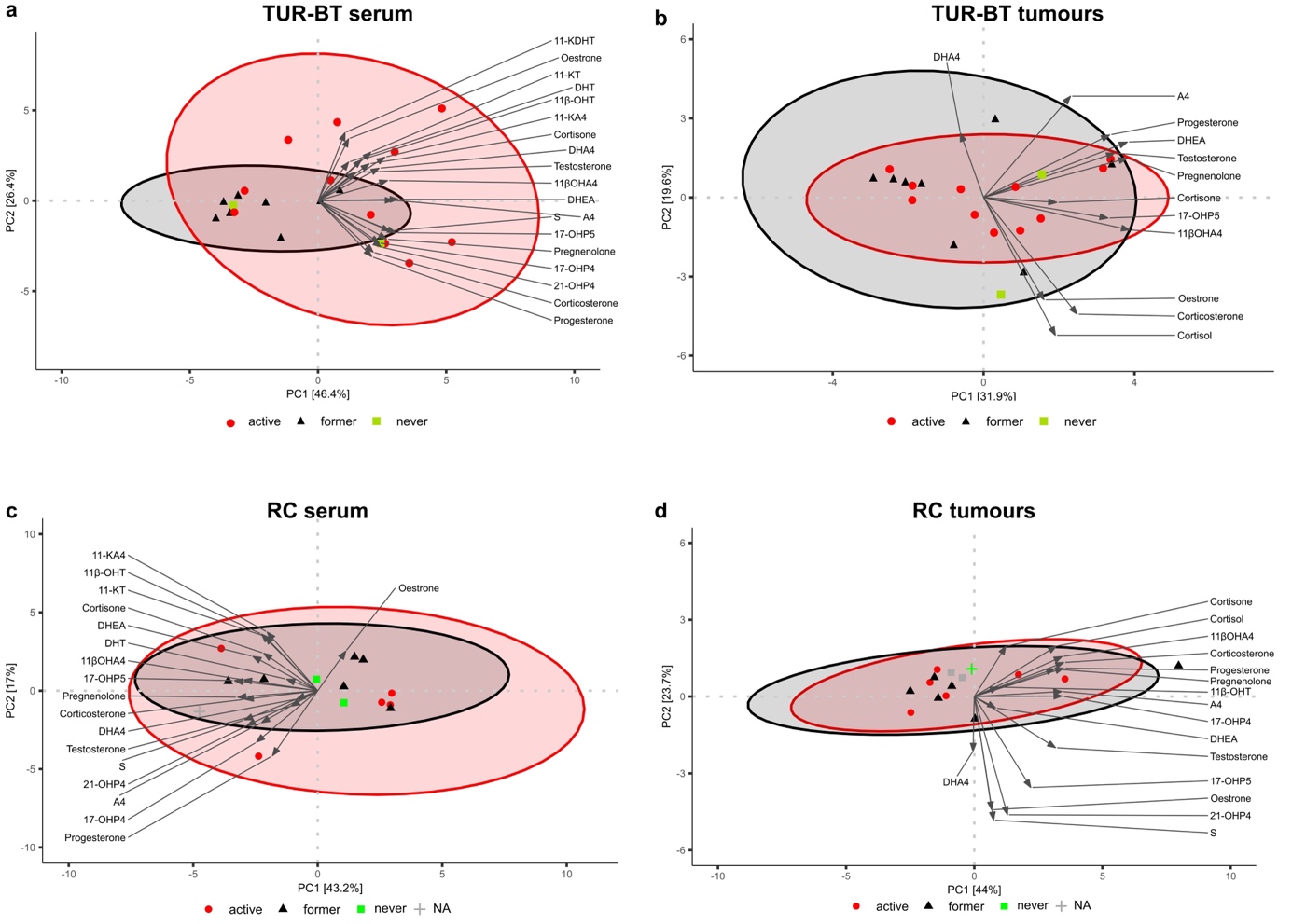


## Supplementary Figure S4

PCA analysis of the samples and smoking status. **a** TUR-BT serum samples **b** TUR-BT tumour samples **c** RC serum samples **d** RC tumour samples.


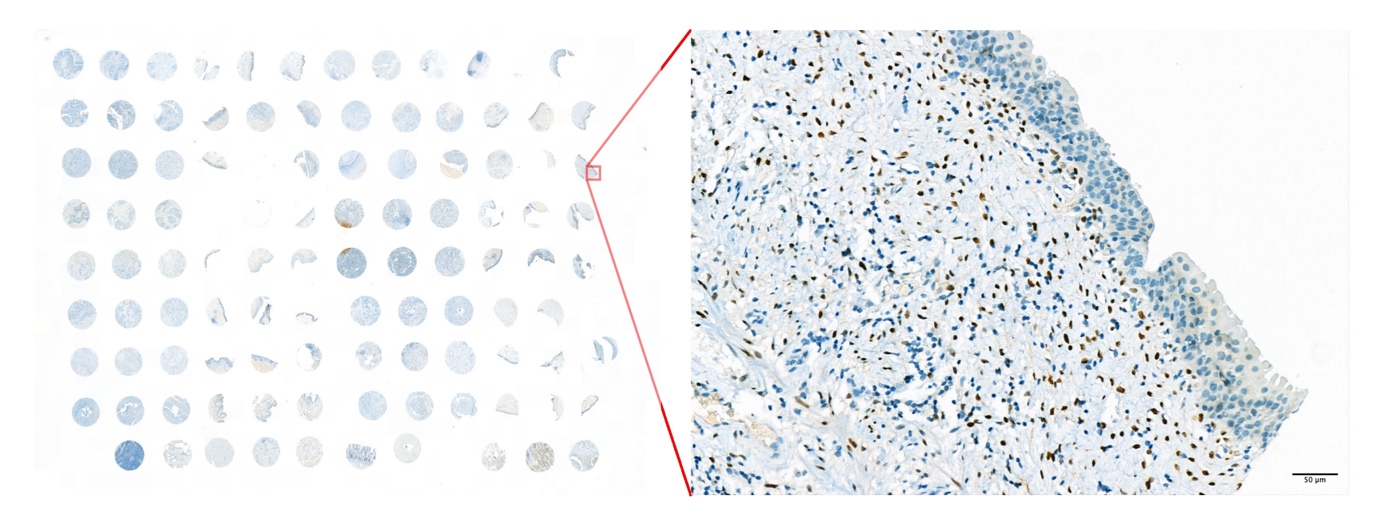


## Supplementary Figure S5

Positive stromal staining of progesterone receptor in IHC.

## Supplementary Figure S6.

Heatmap showing expression of steroid metabolising enzymes and receptors in tumours and adjacent non-tumour tissues.


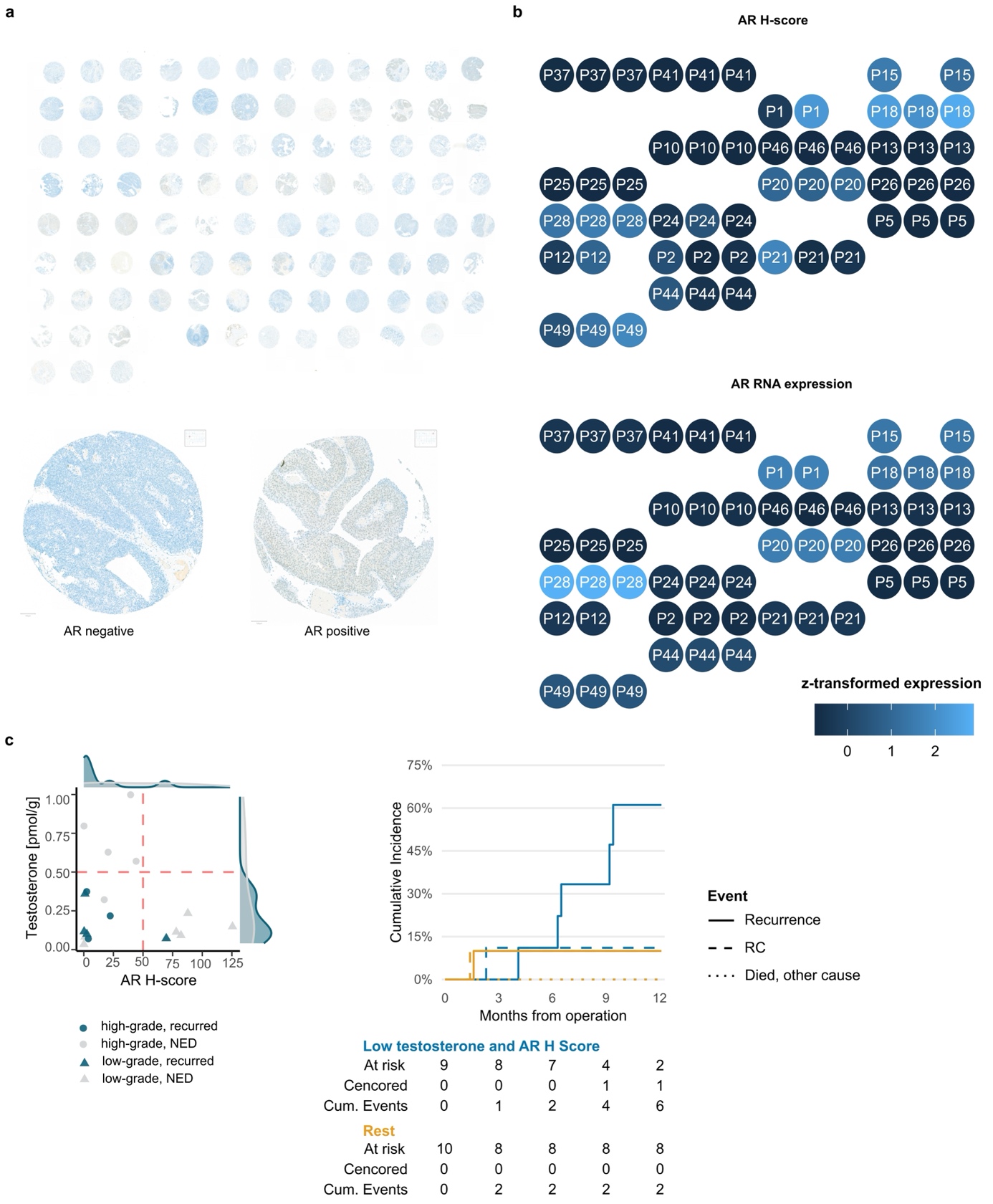


## Supplementary Figure S7.

**a** Top: AR expression of TUR-BT samples in TMA. Bottom: representative examples of AR negative and positive TMA cores, respectively. **b** Top: AR H-scores by TMA cores. Only cores included in the survival analysis are included. Bottom: Corresponding *AR* expression in RNA sequencing. **c** Left: plot showing the association between intratumoral testosterone levels and AR H-score. Right: Cumulative incidence function of low testosterone and AR H-score group vs rest.

## Supplementary Figure S8.

PCA analysis of serum steroid levels between patients with MIBC or NMIBC. The green ellipse is estimated by all NMIBC samples. NED: No evidence of disease.

## Supplementary Figure S9.

60-month follow-up of steroid-hot and steroid-cold groups.

## Supplementary Figure S10.

60-month follow-up of low testosterone and AR expression vs rest.
